# Modeling Stromal Cells Inside the Tumor Microenvironment of Ovarian Cancer: In Vitro Generation of Cancer‐Associated Fibroblast‐Like Cells and Their Impact in a 3D Model

**DOI:** 10.1002/mco2.70172

**Published:** 2025-04-17

**Authors:** Jacopo Romoli, Paola Chiodelli, Patrizia Bonassi Signoroni, Elsa Vertua, Clarissa Ferrari, Elisabetta Giuzzi, Alice Paini, Elisa Scalvini, Andrea Papait, Francesca Romana Stefani, Antonietta Rosa Silini, Ornella Parolini

**Affiliations:** ^1^ Department of Life Science and Public Health Università Cattolica del Sacro Cuore Rome Italy; ^2^ Centro di Ricerca E. Menni Fondazione Poliambulanza Istituto Ospedaliero Brescia Italy; ^3^ Research and Clinical Trials Unit Fondazione Poliambulanza Istituto Ospedaliero Brescia Italy; ^4^ Fondazione Policlinico Universitario “Agostino Gemelli” IRCCS Rome Italy

**Keywords:** 3D model, cancer‐associated fibroblast, ovarian cancer, tumor microenvironment

## Abstract

The tumor microenvironment (TME) is the combination of cells and factors that promotes tumor progression, and cancer‐associated fibroblasts (CAFs) are a key component within TME. CAF originates from various stromal cells and is activated by factors such as transforming growth factor‐beta (TGF‐β) secreted by tumor cells, favoring chemoresistance and metastasis. Recent publications have underlined plasticity and heterogeneity and their strong contribution to the reactive stroma within the TME. Our study aimed to replicate the TME's structure by creating a 3D in vitro model of ovarian cancer (OC). By incorporating diverse tumor and stromal cells, we simulated a physiologically relevant environment for studying CAF‐like cell behavior within tumor spheroids in a context‐dependent manner. CAF‐like cells were generated by exposing human dermal fibroblasts to OC cell line conditioned media in the presence or absence of TGF‐β. Herein, we found that different stimuli induce the generation of heterogeneous populations of CAF‐like cells. Notably, we observed the ability of CAF‐like cells to shape the intratumoral architecture and to contribute to functional changes in tumor cell behavior. This study highlights the importance of precise assessment of CAF for potential therapeutic interventions and further provides a reliable model for investigating novel therapeutic targets in OC.

## Graphical abstract

Conditioned medium from ovarian cancer cell lines influences fibroblast differentiation into distinct CAF subpopulations (iCAF, myCAF). A 3D model mimicking the tumor microenvironment was developed to study CAF roles in tumor spheroid growth and invasion. Results reveal tumor‐driven fibroblast reprogramming and offer a dependable framework for exploring new treatment targets in ovarian cancer.

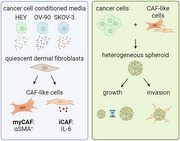

## 1 Introduction

In the last two decades, the scientific community has extensively studied and reinforced the concept of tumor microenvironment (TME). The TME constitutes the cells, factors, and components, such as extracellular matrix (ECM) and vessels, which characterize the tumor mass composition and architecture. Within this dynamic environment, stromal cells, including cancer‐associated fibroblasts (CAF) [1, 2], interact with tumor cells as the tumor grows, leading to a mutual influence that forms the basis for TME development. CAF has been shown to have a fundamental role in determining tumor progression. First, they help cancer cells escape from the immune system [3] by releasing specific factors and cytokines and display a marked capacity to invade surrounding tissues and contribute to metastasis, thus increasing tumor aggressiveness and progression [4, 5, 6]. Secondly, CAF also exhibits the capacity to limit drug efficacy by decreasing drug penetration inside the tumor mass. Indeed, abundant ECM deposition by CAF favors chemoresistance mechanisms and hinders immune cell infiltration [7, 8].

Since a specific marker to uniquely identify CAF is lacking, a combination of markers is typically employed to detect their activation shift. A recent consensus statement from experts in CAF biology outlines common criteria for identifying CAF, including negativity for epithelial, endothelial, and lymphocytic markers (E‐cadherin, CD31, CD45) and positivity for fibroblast activation markers such as α‐smooth muscle actin (αSMA) and fibroblast activation protein (FAP) [1]. Additionally, proteins involved in ECM modification, such as collagen, tenascin C, metalloproteinases (MMPs), and tissue inhibitors of metalloproteinases (TIMPs), are now considered CAF‐related markers [2]. Based on this understanding, CAFs are identified by the combined expression of TGF‐β, αSMA, and functional assessment [1, 2, 3, 4, 5, 6, 7, 8, 9].

CAF phenotypes differ across tumor types, leading to the identification of various CAF subtypes. Innovative techniques like single‐cell technologies have reinforced our understanding of CAF heterogeneity, enabling the first profiling of major subpopulations and recognizing their uniqueness in each tumor [10, 11, 12]. Considering their distinct phenotypes, functional properties, and spatial distribution inside the TME, to date, three categories of CAF have been characterized: myofibroblast‐like CAF (myCAF), inflammatory CAF (iCAF), and antigen‐presenting CAF (apCAF). myCAF are the most prominent subpopulation of stromal cells in the TME and are mainly localized close to the tumor [13]. myCAF has been characterized for the expression of cytoskeletal proteins such as αSMA in combination with TGF‐β expression [14, 15, 16]. iCAF is located at the periphery of the tumor and is characterized by the expression of large amounts of inflammatory mediators and chemokines (i.e., IL‐6), thus chronic inflammation and an immunocompetent environment, a hallmark of cancer [17, 18]. Lastly, apCAF are the most recent subpopulation of fibroblasts discovered in the TME [18] and are considered to be immunosuppressive, although their properties are yet to be defined. According to tumor type, CAF subpopulations can vary in terms of secreted factors and activated state, affecting the angiogenetic process [19], stemness, and self‐renewal of different cancer types [20, 21, 22, 23, 24, 25, 26]. In this study, we focused on ovarian cancer (OC) due to the highly heterogeneous nature of its reactive stroma. OC is the eighth most common cause of death from cancer among women worldwide [27]. OC harbors different subtypes of CAF (namely S1, S2, S3, and S4) that are distinguished based on the expression of αSMA, FAP, and CD29 or based on the analogies with cancer cells in terms of gene expression profile [28, 29]. In OC, also podoplanin (PDPN) and microfibril‐associated protein 5 (MFAP5) have been recently identified as specific markers of CAF [30, 31], the latter being implicated in the pathological behavior of CAF within the OC microenvironment by providing input for collagen synthesis [32]. CAF has been shown to contribute to OC malignant behavior by (i) increasing the migration capacity of cancer cells isolated from normal ovary tissues in vitro [33], (ii) promoting the formation of ascitic multicellular aggregates (MCAs), namely cellular spheroids, which in turn favor peritoneal metastasis [34, 35], and (iii) enhancing chemoresistance and the generation of a stem‐like phenotype in high‐grade serous ovarian carcinoma (HGSOC) [36, 37].

However, despite advances, ex vivo modeling of the TME in OC remains challenging, and the spatial and temporal dynamics of cells within OC heterogeneous spheroids have not been fully explored. The aim of this study was to generate phenotypically and functionally relevant CAF‐like cells in vitro by exposing human dermal fibroblasts to the secreted factors of different types of OC cell lines and to the cytokine TGF‐β.

Herein, we demonstrated that ovarian OC lines with a diverse background differentially influence human fibroblast fate determination. Further, we generated a highly relevant 3D model by combining tumor cells and CAF‐like cells to mimic the dimensional structure of the TME in vivo and to specifically address the role of CAF in tumor spheroid growth and invasion capacity.

Our model represents a highly relevant in vitro model that mimics the structural organization of the TME. Our model provides a platform that is both controlled and reproducible, ensuring ethical appropriateness for high‐throughput screening and detailed analysis. Through the utilization of this model, researchers can explore the complex interactions between tumors and the stroma, opening avenues for the advancement of innovative personalized medicine strategies that target the TME.

## 2 Results

### 2.1 Human Dermal Fibroblasts Exposed to Conditioned Media From OC Cells Acquire a Sustained CAF‐Like Phenotype

**FIGURE 1:**
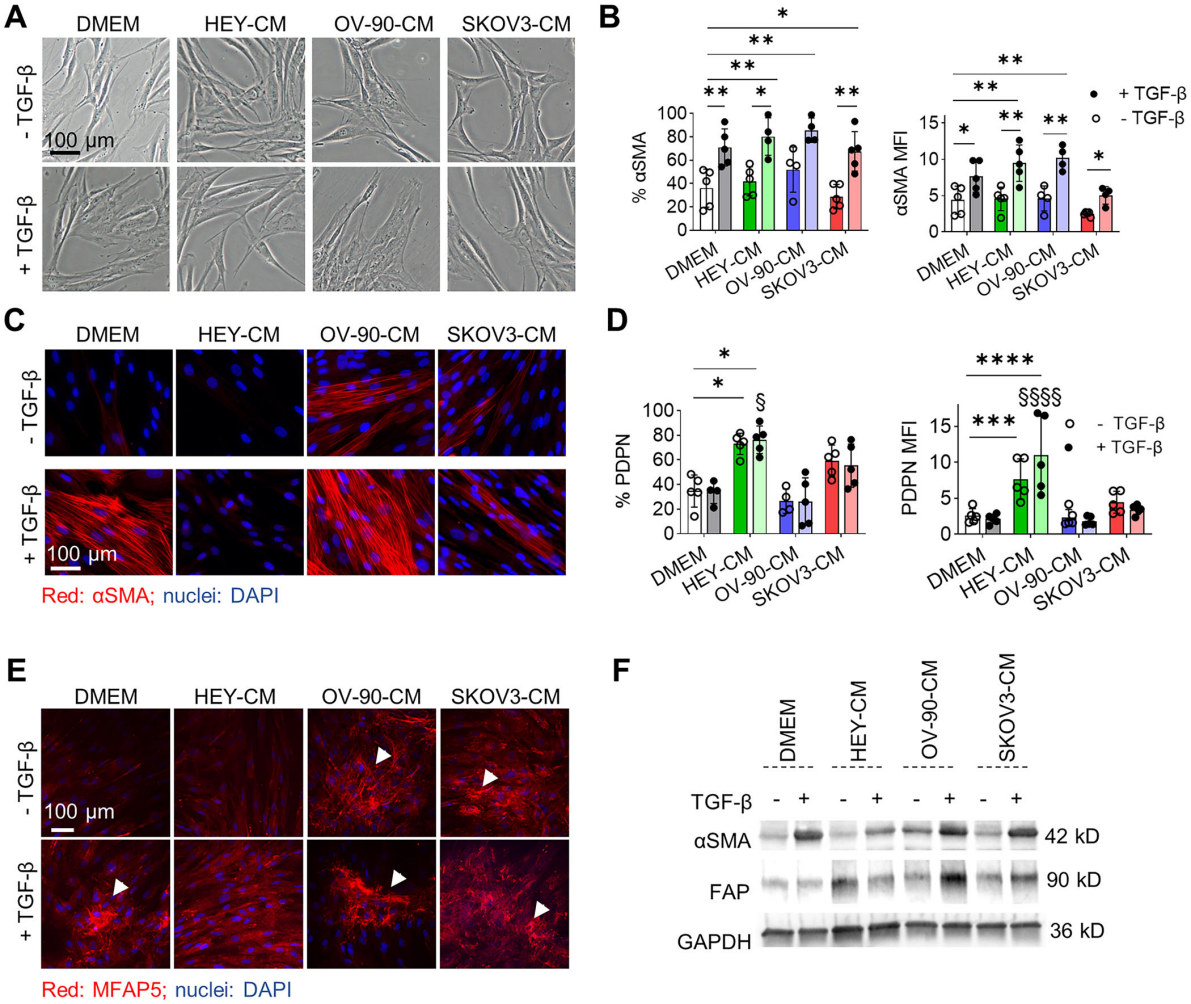
Ovarian cancer cell‐conditioned medium and TGF‐β induce CAF‐related protein expression. Dermal fibroblasts were exposed to HEY‐CM, OV‐90‐CM, and SKOV3‐CM in the absence or presence of TGF‐β for 7 days. (A) Representative images of fibroblasts cultured under different conditions for 7 days. Scale bar = 100 µm. (B) αSMA protein expression evaluated by flow cytometry. The results show the percentage of αSMA^+^ cells and the relative mean fluorescence intensity (MFI). *n* = 4–5. (C) Cells were stained with αSMA antibody (red), and nuclei were counterstained with DAPI (blue). Pictures were acquired at 63× magnification (scale bar 100 µm). (D) PDPN protein expression was evaluated by flow cytometry. The results show the percentage of PDPN^+^ cells and the relative MFI. *n* = 4–5. (E) Cells were stained with MFAP5 antibodies (red), and nuclei were counterstained with DAPI (blue). White arrowheads indicate MFAP5 deposition. Pictures were acquired at 20× magnification, scale bar 100 µm. (F) Cell lysates were analyzed by western blot with αSMA and FAP antibodies and normalized for GAPDH. Molecular weights of the respective bands, expressed in kDa, are shown on the right. *p < 0.05; **p < 0.01; ***p < 0.001; ****p < 0.0001 treatments versus TGF‐β. ^§^ *p* < 0.05; ^§§§§^ *p* < 0.0001 treatments versus DMEM + TGF‐β.

We first interrogated the influence of tumor cell secretome (hereafter collectively referred to as OC cell conditioned media [CM], OC‐CM) on human dermal fibroblasts and, in particular, on their activation status. Dermal fibroblasts were exposed to one of the three OC‐CM (HEY‐CM, OV‐90‐CM, or SKOV3‐CM) either alone or in the presence of exogenous TGF‐β, a cytokine known to play a prominent role in CAF activation [38]. After a 7‐day treatment, changes in morphology and marker expression were evaluated. TGF‐β alone or in the presence of OC‐CM induced morphological changes in normal fibroblasts. Indeed, fibroblasts, which have a characteristic spindle‐shaped morphology, became flattened and acquired αSMA positivity, especially those treated with OV‐90‐CM, which further acquired a more compact shape (Figure 1A). Flow cytometry analysis showed that the percentage of αSMA^+^ cells increased in the presence of TGF‐β or OV‐90‐CM, while HEY‐CM or SKOV3‐CM alone had no effect when compared to the control condition (DMEM). TGF‐β increased the percentage of αSMA^+^ cells when added to OC‐CM; these observations are corroborated in part also by MFI analysis (Figure 1B). Extending from these findings and given that αSMA protein undergoes structural changes in activated fibroblasts, we performed morphological analyses to investigate the effects of OC‐CM on fibroblast activation (Figure 1C). We confirmed the ability of TGF‐β to induce αSMA organization and observed a similar pattern for OV‐90‐CM. Indeed, OV‐90‐CM induces morphological changes in αSMA, thus supporting the data obtained by flow cytometry analysis and the morphological changes observed in Figure 1A. Contrarily, HEY‐CM and SKOV3‐CM alone were unable to modify αSMA organization, and the cells maintained their classic spindle‐shaped morphology. On Day 3 (Figure S1A), and more prominently on Day 7, both HEY‐CM and SKOV3‐CM significantly increased the number of PDPN^+^ cells (Figure 1D). The addition of TGF‐β did not have any additional effect. Neither TGF‐β nor OV‐90‐CM induced an increase in PDPN^+^ cells. Also, human adult dermal fibroblasts were able to respond in a similar way to the different OC‐CM, even if with a generally lower expression of αSMA (Figure S2). To further investigate the CAF profile, we next sought to analyze MFAP5, a protein implicated in the pathological behavior of CAF within the OC microenvironment [32]. MFAP5 is released by CAF and binds integrins [32], thus providing input for collagen synthesis. It can also bind fibrillin 2 in the ECM and accumulate in the extracellular space [39], forming the fibrillary structures shown in Figure 1E (white arrows). All treatments except HEY‐CM led to MFAP5 deposition and the generation of fibrillar structures. Furthermore, a similar trend of increase in protein expression was confirmed by western blot analysis for αSMA, fibroblast‐activated protein (FAP), another classical CAF marker, and MFAP5 (Figures 1F and S1B,C).

**FIGURE 2:**
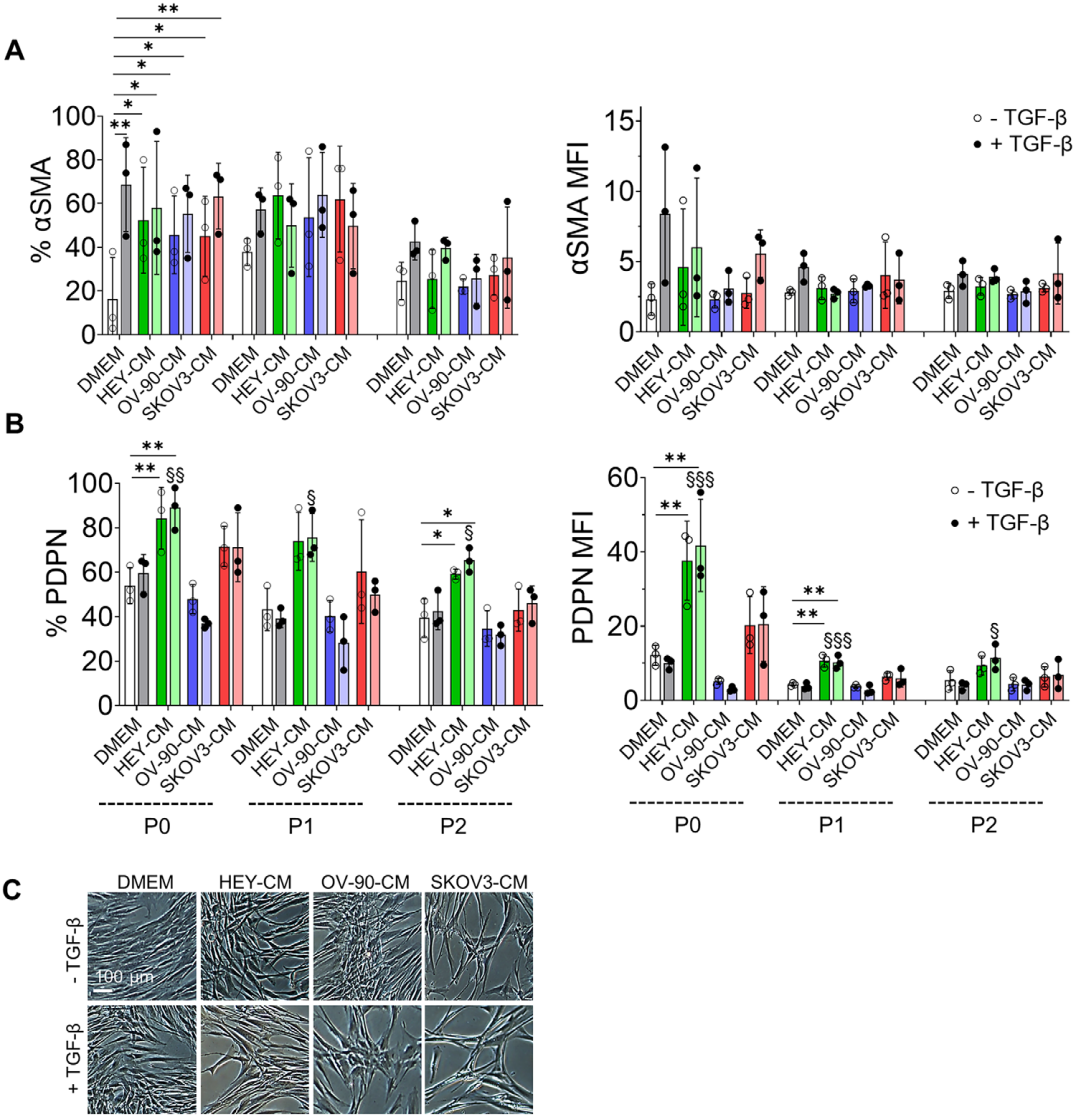
CAF‐like cell markers and morphology over time. Dermal fibroblasts were exposed to HEY‐CM, OV‐90‐CM, and SKOV3‐CM in the absence or presence of TGF‐β for 7 days (P0). The cells were cultured without CM until passage 2 (P2), then αSMA (A) and PDPN (B) markers were evaluated using flow cytometry. The percentage of αSMA^+^ and PDPN^+^ cells and the relative mean fluorescence intensity (MFI) were determined. *n* = 3–4. *p < 0.05; ** *p* < 0.01; treatments versus TGF‐β. ^§^ *p* < 0.05; ^§§^ *p* < 0.01; ^§§§^ *p* < 0.001; treatments versus DMEM + TGF‐β. (C) Representative images of fibroblasts cultured under‐reported conditions and treatments at P1. Scale bar 100 µm.

To assess whether activated fibroblasts could maintain their phenotype over time, 7 days after the different treatments, CAF‐like cells were cultured in DMEM until passage 2 (P2) or alternatively frozen, thawed, and cultured in DMEM for 3 days. The samples were then analyzed using flow cytometry for the expression of αSMA and PDPN. For αSMA, the expression levels were lower compared to those observed after 7 days of culture with the treatments (Figures 2A and S3A). As shown in Figures 2B and S3B, treatment with HEY‐CM and SKOV‐CM, even in the presence of TGF‐β, maintains the percentages of PDPN^+^ cells both after trypsinization and maintenance in DMEM until P1 and, to a lesser extent at P2, as well as after freezing and thawing. Note that the cells at P1 maintain the morphological features acquired with the treatment, as reported in Figures 2C and S3C.

In summary, our results suggest that TGF‐β and OV‐90‐CM have similar effects on αSMA cytoskeletal organization and that both OV‐90‐CM and SKOV3‐CM regulate MFAP5 structures, while HEY‐CM and SKOV‐CM enhance PDPN expression. Furthermore, they maintain PDPN positivity for expression both after the freezing‐thawing procedure and after passaging, while the αSMA percentage decreased in both procedures.

### 2.2 Treated Fibroblasts Acquire Different CAF‐Related Functions

**FIGURE 3:**
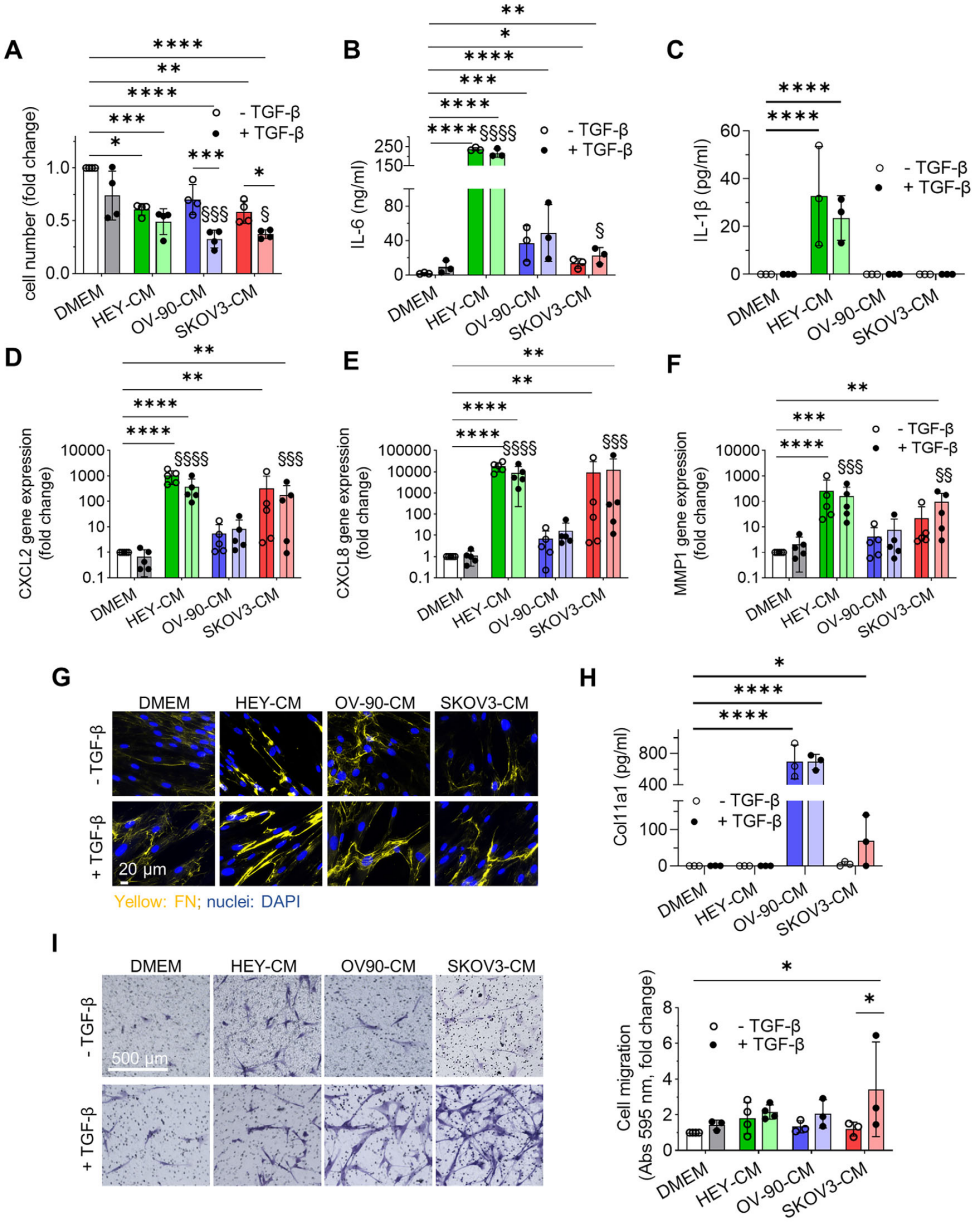
Treated fibroblasts acquire CAF‐related functions: a secretory and functional profile. Dermal fibroblasts were exposed to HEY‐CM, OV‐90‐CM, and SKOV3‐CM in the absence or presence of TGF‐β for 7 days. (A) After 7 days, cells were counted, and data were expressed as the ratio between the different conditions compared to control (DMEM), *n* = 4. (B) The presence of IL‐6 (B) or IL‐1β (C) in the supernatant of the cells cultured in the different conditions for 7 days was assessed by ELISA. Values are reported in nanograms/mL or picograms/mL and were normalized versus basal IL‐6 or IL‐1β in each condition. *n* = 3. (D–F) *CXCL2*, *CXCL8*, and *MMP1* expression was analyzed by RT‐PCR. Changes in gene expression are shown as fold change relative to DMEM treatment, used as a control, on a logarithmic scale. *n* = 5. (G) Cells were stained with FN antibody (yellow), and nuclei were counterstained with DAPI (blue). Pictures were acquired at 63× magnification, scale bar 20 µm. (H) The presence of COL11A1 in the supernatant of the cells cultured in the different conditions for 7 days was assessed by ELISA. Values are reported in picograms/mL and were normalized versus basal COL11A1 in each condition. *n* = 3. (I) Cell migration was analyzed using a transwell assay. In the left panel, representative images of migrated cells; in the right panel, the graph indicates the relative absorbance at 595 nm after crystal violet staining, reported as the ratio between the different conditions compared to control (DMEM). *n* = 3. *p < 0.05; **p < 0.01; ***p < 0.001; ****p < 0.0001; treatments versus TGF‐β. ^§^ *p* < 0.05; ^§§§^ *p* < 0.001; ^§§§§^ *p* < 0.0001; treatments versus DMEM + TGF‐β.

Having shown that dermal fibroblasts treated with OC‐CM and TGF‐β are able to acquire the expression of CAF‐like markers, we next sought to determine whether these cells acquired CAF‐like functions. Fibroblasts treated with OC‐CM showed a decreased proliferation after 7 days of culture in comparison to control. This effect is enhanced by TGF‐β, producing a reduction of their proliferative potential in line with the acquisition of a CAF‐like phenotype [40] (Figure 3A). CAF within the TME can secrete several soluble factors that act in a paracrine or autocrine manner in continuous crosstalk with other tumor components. One of the well‐described cytokines is IL‐6, which is also a marker of the iCAF subpopulation [41]. IL‐6 mRNA expression and protein secretion were evaluated 7 days after exposing cells to different conditions. At the secretory level, TGF‐β increased IL‐6 secretion, consistent with previous findings in human lung fibroblasts [42]. A significant increase in cytokine levels was observed in the supernatant with all OC‐CM, particularly HEY‐CM, and remained high when combined with TGF‐β (Figure 3B). This situation was mirrored by gene expression data (Figure S4A), with the exception of SKOV3‐CM, which induced a high *IL6* expression compared to DMEM. Another factor secreted by CAF in contrast to normal fibroblasts is IL‐1β, known to affect cancer cell invasion. Interestingly, IL‐1β secretion was enhanced only by HEY‐CM when used alone or in combination with TGF‐β (Figure 3C) [43]. Moreover, HEY‐CM and SKOV3‐CM induced a very significant increase of *CXCL8* and *CXCL2* gene expression alone or in the presence of TGF‐β in fibroblasts, suggesting a more pronounced secretory phenotype compared to treatment with OV‐90‐CM (Figure 3D,E). Interestingly, CXCL8 can induce the expression of *MMP1* [44], an expression that, again, we observed pronounced with HEY‐CM and SKOV3‐CM (Figure 3F).

Regarding the ECM, all OC‐CM induced a higher deposition of fibronectin (FN) (Figure 3G), although to a lesser extent when the cells were subjected to SKOV3‐CM treatment. Interestingly, CAF‐like cells showed increased COL11A1 secretion when stimulated with OV‐90‐CM and, to a lesser extent, with SKOV3‐CM (Figure 3H), mirroring the activation of NF‐κB, a transcription factor activated in CAF [45], which was phosphorylated when fibroblasts were stimulated with OV‐90‐CM and SKOV3‐CM (Figure S4B).

On the contrary, *MMP11* and *COL1A1* were downregulated, especially with HEY‐CM and SKOV treatment. Notably, TGF‐β upregulated *COL1A1*, also when combined with OV‐90‐CM (Figure S4C,D).

In addition to the acquisition of the expression of specific CAF‐related markers and a CAF‐related secretory profile, we observed that TGF‐β alone stimulates CAF‐specific functions, including increased migratory ability. Notably, all OC‐CM treatments in the presence of TGF‐β induced a higher migratory capacity in fibroblasts compared to the control (DMEM) and TGF‐β alone, as assessed by transwell assay (Figure 3I) and by wound healing assay (Figure S4G).

### 2.3 Tumor Cell Secretome Differentially Influences the Expression of CAF‐Related Genes

**FIGURE 4:**
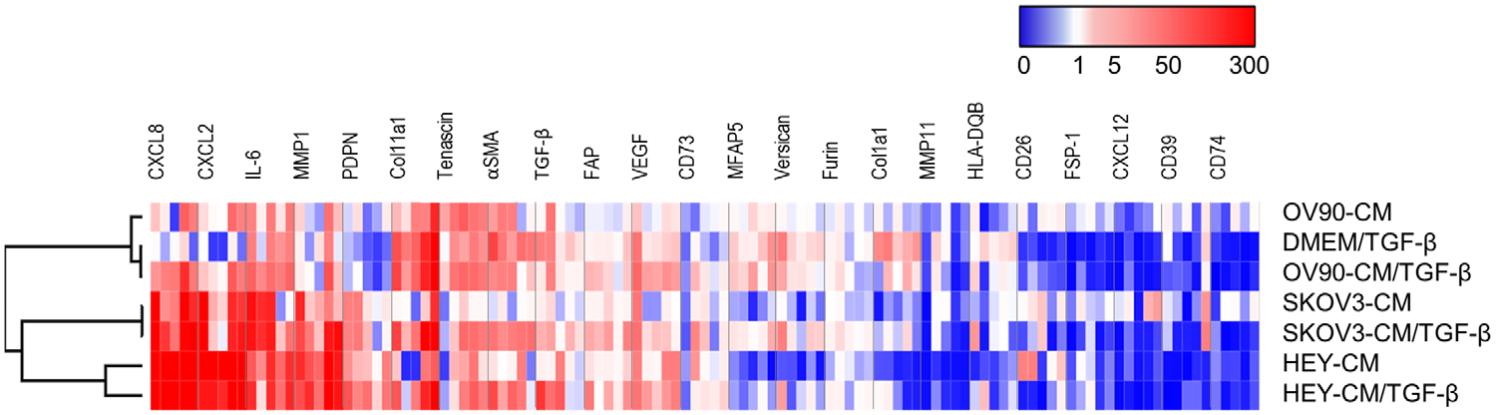
Expression pattern of CAF‐related genes following different treatments. Fibroblasts from five donors were exposed to HEY‐CM, OV‐90‐CM, and SKOV3‐CM in the absence or presence of TGF‐β for 7 days. RT‐PCR and gene expression analysis were performed on 15 genes (upper part of the graph). The results are expressed as a fold change in comparison to the DMEM (control). On the left, the clustering obtained by using the “MORFEUS” online tool is represented. Blue squares represent the downregulated genes, white squares unchanged genes, and red squares upregulated genes (scale bar).

To evaluate the influence of the different tumor‐derived cell lines and of TGF‐β on CAF‐like features, we analyzed the gene expression profile by RT‐PCR of dermal fibroblasts 7 days after treatment, comparing the different expression levels to the control condition (DMEM) (Figure 4). We investigated 15 genes that are related to the CAF phenotype and focused on genes that were upregulated in OC studies [46, 47, 48].

Gene expression analysis was performed by using a generalized linear mixed model with fixed effects: genes, conditions, and their interaction (gene × conditions). The results indicate that the gene expression values are statistically different (*p* < 0.001) between the genes, with the most “abundant” genes being *CXCL8*, *CXCL2*, and *COL11A1*. The conditions also showed statistically different values: DMEM/TGF‐β and OV‐90‐CM are the ones that changed the least, with lower abundance. CM‐SKOV3 and HEY‐CM (with and without TGF‐β) are the ones that changed the most. The interaction effect was significant (*p *< 0.001), indicating that the gene abundance varied between the conditions. Specifically, *CXCL8* was the most abundant in HEY‐CM and SKOV3‐CM (with and without TGF‐β), while *CXCL2* was the most abundant in HEY‐CM and in HEY‐CM+TGF‐β (as seen in Figure 3D,E). *COL11A1* was the most abundant in OV‐90‐CM and OV‐90‐CM + TGF‐β (Figure S5). We found that the expression of the genes encoding IL‐6 (*IL6*), COL11A1 (*COL11A1*), Tenascin (*TNC*), αSMA (*ACTA2*), TGF‐β (*TGFB1*), FAP (*FAP*), PDPN (*PDPN*), versican (*VCAN*), and furin (*FURIN*) was the highest in fibroblasts treated with OC‐CM/TGF‐β compared to the others, presenting some similarities with DMEM/TGF‐β. In particular, the matrix shows that HEY‐CM and HEY‐CM/TGF‐β, SKOV‐3, and SKOV3‐CM/TGF‐β are the conditions presenting the main shared gene‐expression profile. We observed that the expression of the genes encoding CD26 (*DPP4*), FSP‐1 (*S100A4*), CXCL12 (*CXCL12*), and CD39 (*ENDTPD1*) was downmodulated in the presence of TGF‐β (Figure 4). In summary, the results indicate statistically significant differences in gene expression according to the different conditions, as well as an interaction effect where the most abundant gene varies depending on the specific condition.

Our findings on dermal fibroblast treatments suggest distinct gene expression patterns associated with different treatments that promote a CAF‐like phenotype.

### 2.4 CAF‐Like Cells Influence Heterogeneous Spheroid Growth

**FIGURE 5:**
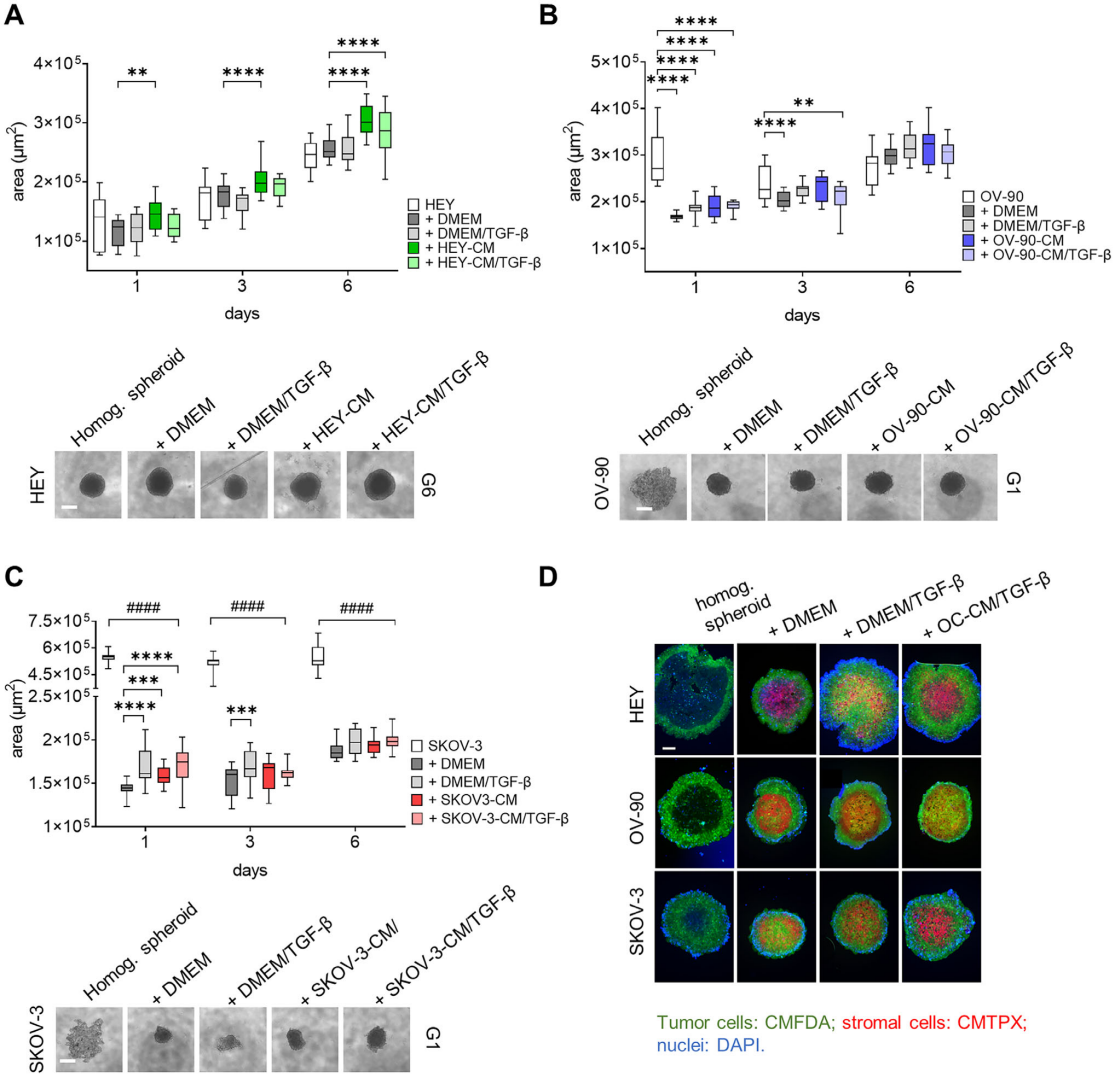
Characterization of heterogeneous spheroids. HEY (A), OV‐90 (B), and SKOV3 (C) tumor cells were either grown as homogeneous (when alone) or heterogeneous (when in the presence of CAF‐like cells) spheroids, and their growth was monitored for 6 days. Area was measured at Days 1, 3, and 6 after spheroid generation. The area was measured in µm [2]. On the bottom, representative microphotographs of spheroids on Day 6 for HEY (A) and Day 1 for OV‐90 (B) and SKOV3 (C) (scale bar 200 µm). Homogeneous spheroids are shown with white blocks; the representation of the heterogeneous spheroids with CAF‐like cells in different conditions is shown as follows: DMEM grey blocks; DMEM/TGF‐β light grey blocks; OC‐CM colored blocks; and OC‐CM/TGF‐β as light‐colored blocks (green for HEY‐CM, blue for OV‐90‐CM, orange for SKOV3‐CM); *n* = 3–4. *p < 0.05; **p < 0.01; ***p < 0.001; ****p < 0.0001. ^####^ *p* < 0.0001 all treatments versus DMEM. (D) Representative immunofluorescence images of homogeneous spheroids generated with tumor cells (green, CMFDA) and heterogeneous spheroids generated with tumor (green, CMFDA) and stromal cells (red, CMTPX). Nuclei are counterstained with DAPI (blue). Pictures were acquired at 4× magnification. Scale bar 100 µm.

The CAF function is strictly related to the TME. To address the behavior of CAF‐like cells within a complex environment, we generated OC heterogeneous spheroids and evaluated cellular dynamics and interplay involved in cancer progression. Heterogeneous spheroids composed of the different tumor cell lines and dermal fibroblasts preconditioned with different stimuli (TGF‐β, OC‐CM±TGF‐β, as described above) were photographed at different time points, and their area was measured. Interestingly, all heterogeneous spheroids show a more robust increase in area when compared to homogeneous HEY spheroids (Figure 5A), and starting from Day 3, the presence of CAF‐like cells increases the area of heterogeneous spheroids with respect to those composed of only tumor cells. Moreover, from Days 1 to 6 of coculture, heterogeneous spheroids composed of HEY‐CM or HEY‐CM/TGF‐β CAF‐like cells are significantly bigger than those composed of control fibroblasts. Contrarily, the addition of the stromal component resulted in more compact spheroids derived from OV‐90 and SKOV3 cell lines (see microphotographs in Figure 5B,C). During the 6‐day culture period, the area of homogeneous spheroids decreased from Days 1 to 3 and then began to slightly increase on Day 6. In contrast, heterogeneous spheroids were more compact on Day 1 and steadily increased in size until Day 6, exhibiting a larger area regardless of the presence of stromal cells. SKOV3 homogeneous spheroids displayed a behavior similar to that of OV‐90 spheroids, with a decrease in area from Days 1 to 3 that remained constant until Day 6 (Figure 5B). On Day 1, the area of SKOV3 heterogeneous spheroids was lower due to their more compact state. The area increased in the presence of different fibroblasts, particularly with TGF‐β or SKOV3‐CM+TGF‐β pretreatment when compared to fibroblasts cultured in DMEM (Figure 5C). Finally, in order to better visualize the biodistribution of stromal cells and tumor cells within the spheroids, cells were differentially labeled. Although with some differences in density and homogeneity, stromal cells (red) generally localized at the center of the spheroid, while tumor cells (green) are found at the edge (Figure 5D). This conformation resembles that observed in HGSOC‐derived ascitic spheroids, where epithelial cells surround a core of CAF [49].

### 2.5 Different OC‐CM Induce Material Transfer From CAF‐Like Cells Toward Tumor Cells and Affect CAF‐Like Cells Phenotype

**FIGURE 6:**
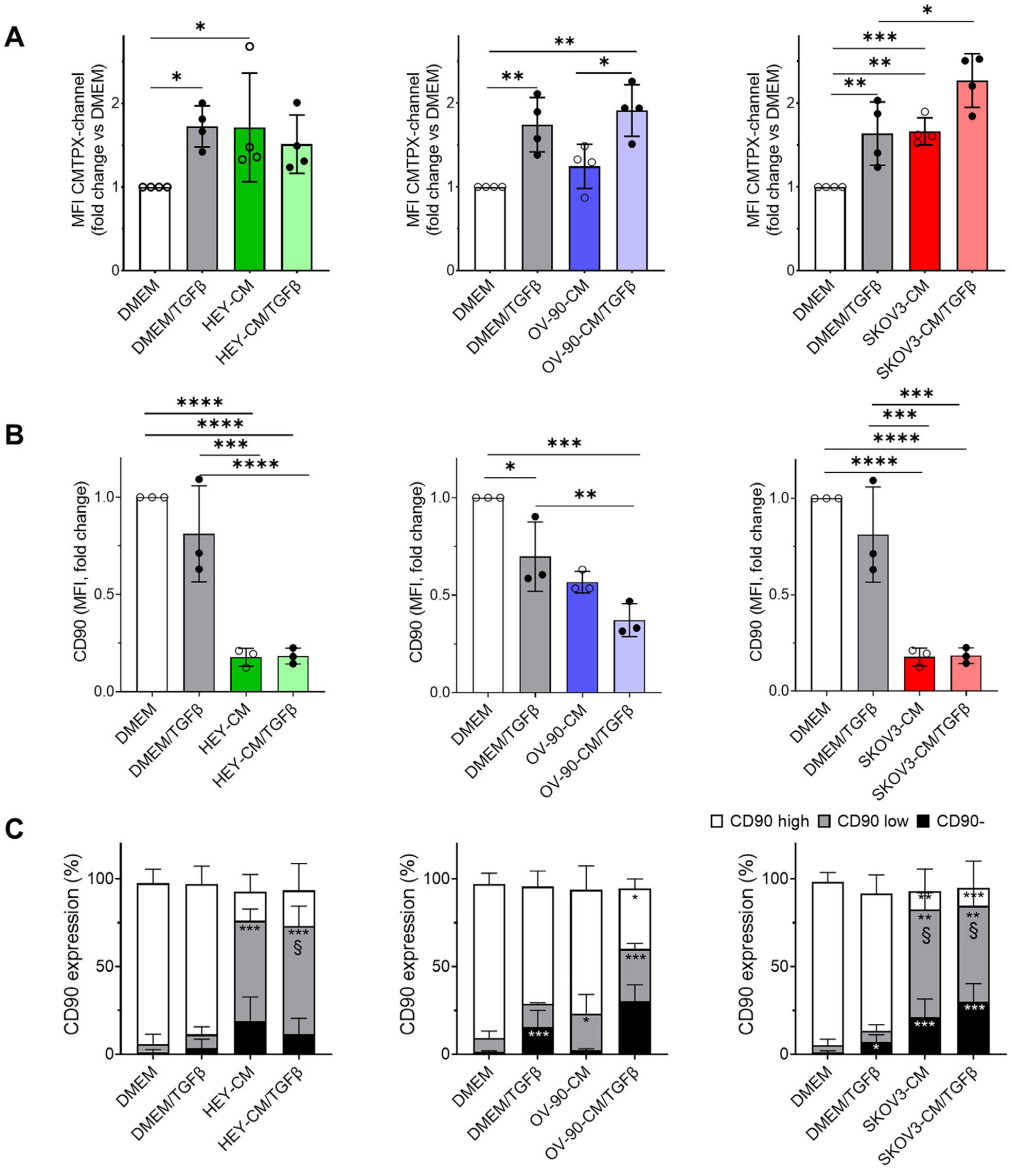
Characterization of CD90 expression on CAF‐like cells in heterogeneous spheroids. (A) Quantification of the MFI at 24 h for the CMTPX channel in unstained tumor cells from heterogeneous 24 h spheroids. *n* = 4. (B, C) Quantification of CD90 expression on fibroblasts and CAF‐like cells in heterogeneous spheroids at 24 h. (B) MFI of CD90 positive cells and (C) percentage of CD90 negative (CD90−), and CD90 low and CD90 high fibroblasts and CAF‐like cells. *n* = 3. *p < 0.05; **p < 0.01; ***p < 0.001, ****p < 0.0001. In Panel C, ^§^ *p* < 0.05; treatments versus DMEM + TGF‐β.

The oncogenic potential of extracellular vesicles (EVs) derived from CAF has been attributed to the transfer of proteins and noncoding RNAs, which can induce epithelial–mesenchymal transition (EMT), invasion, metastasis, and proliferation in recipient cancer cells [50, 51]. In this study, we employed a fluorescent probe, CMTPX, to assess the transfer of material from fluorescently labeled CAF‐like cells (donor cells) to tumor cells (recipient cells). We observed a significant shift in the CMTPX MFI peak of tumor cells in heterogeneous spheroids after 24 h of coculturing with CAF‐like cells compared to fibroblasts cultured in DMEM (Figure 6A). This effect was particularly pronounced when the fibroblasts were derived from OC‐CM/TGF‐β treatments, indicating a more extensive transfer of materials to tumor cells in the presence of CAF‐like cells.

To further characterize the stromal counterpart, we analyzed changes in CD90 expression by flow cytometry analysis on the CMTPX‐positive fraction that identifies all the stromal components of the heterogeneous spheroids. As depicted in Figure 6B, our results revealed a decrease in CD90 expression, as measured by MFI, in CAF‐like cells derived from OC‐CM, particularly in the presence of TGF‐β. We identified three distinct populations based on CD90 expression: CD90 negative, CD90 low, and CD90 high. Among them, CAF‐like cells derived from OC‐CM exhibited an increased CD90 negative fraction and an elevated CD90 low population, particularly after HEY‐CM and SKOV3‐CM, both with and without TGF‐β (Figure 6C). Taken together, these data suggest that OC‐CM treatment significantly affects CD90 expression in stromal cells.

### 2.6 CAF‐Like Cells Induce Spheroid Sprouting

**FIGURE 7:**
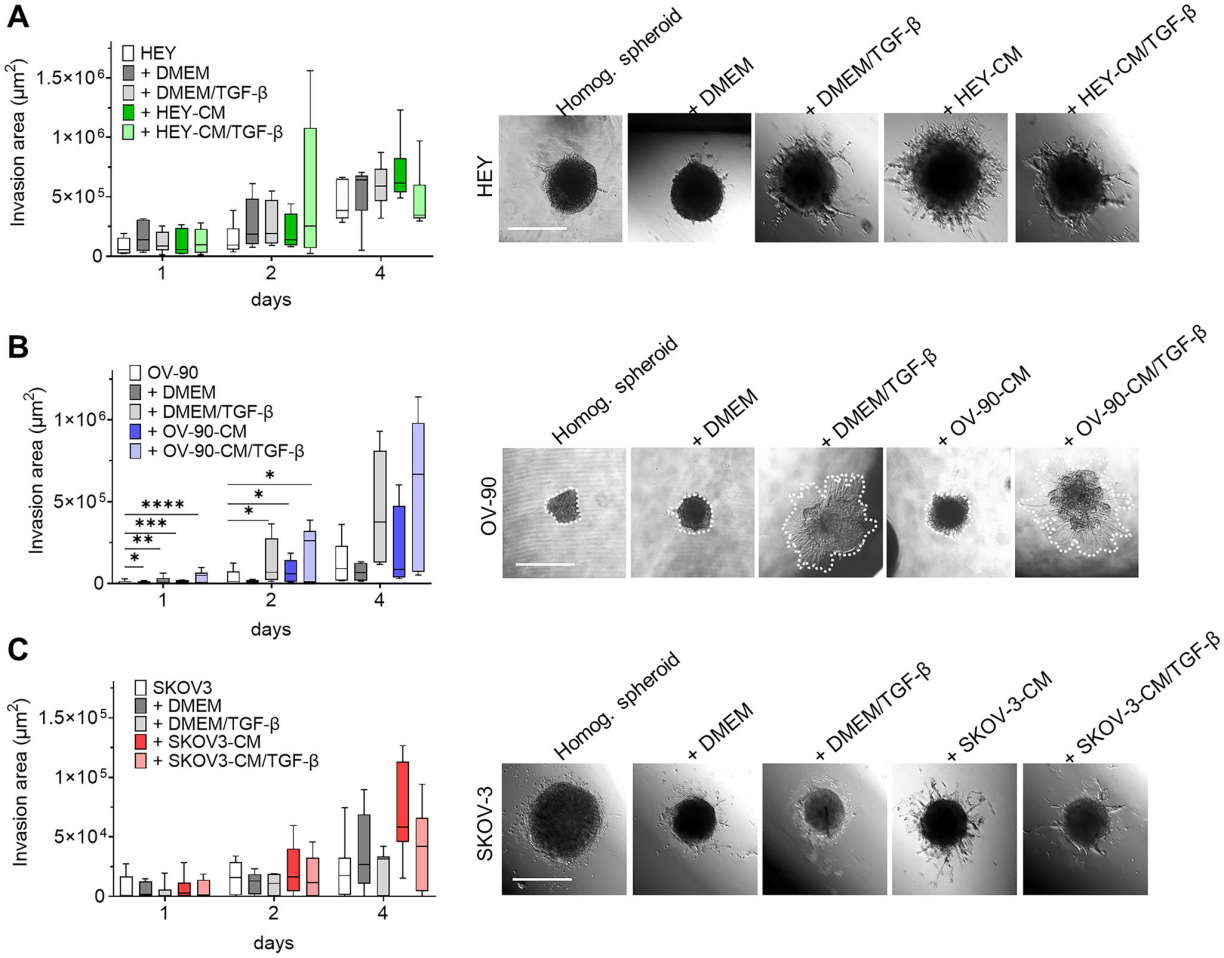
CAF‐like cells increase spheroid invasion. The invasion ability of homogeneous and heterogeneous spheroids generated with HEY (A), OV‐90 (B), and SKOV3 (C) cells was measured for 4 days. The reported values consider the difference between the growing branches and the inner mass. Homogeneous spheroids are shown with white blocks; the representation of the heterogeneous spheroids with CAF‐like cells in different conditions is shown as follows: DMEM grey blocks; DMEM/TGF‐β light grey blocks; OC‐CM colored blocks; and OC‐CM/TGF‐β light‐colored blocks (green for HEY‐CM, blue for OV‐90‐CM, and orange for SKOV3‐CM). *n* = 3–4. On the right, representative spheroid microphotographs of indicated conditions at Day 2 (HEY) or Day 4 (OV‐90 and SKOV3). The invasion area is indicated with a white dotted line for OV‐90. Scale bar 500 µm. *p < 0.05; **p < 0.01; ***p < 0.001; ****p < 0.0001.

Invasion of surrounding normal tissues is widely regarded as a key characteristic of malignant tumors. CAF plays a crucial role in this process due to their ability to contract ECM and facilitate the invasion of cancer cells. We, therefore, investigated the capacity of heterotopic spheroids to invade the surrounding matrix by monitoring their capacity to generate sprouts once seeded on a Geltrex‐coated well. We found that the ability of HEY spheroids to invade the surrounding matrix increased in the presence of stromal cells. Interestingly, on Day 2, heterogeneous spheroids formed by CAF‐like cells from HEY‐CM + TGF‐β treatment showed the most marked increase in invasive areas compared to other heterogeneous spheroids. This value was reversed at Day 4 due to the dynamics of these invasive structures, which are in continuous remodeling and may undergo resorption. On the other hand, spheroids made with CAF‐like cells from HEY‐CM showed the highest values on Day 4 (Figure 7A) compared with fibroblasts cultured in DMEM ± TGF‐β. Heterogeneous OV‐90 spheroids did not display a visible invasive ability, and this was also observed when OV‐90 cells were added to fibroblasts cultured in DMEM. Interestingly, when fibroblasts were pretreated with TGF‐β or OV‐90‐CM + TGF‐β, the invasion ability was increased (Figure 7B). Finally, SKOV3 spheroids showed limited invasion ability when cultured in the absence of stromal cells. The addition of stromal cells did not alter the sprouting ability. In contrast, the addition of CAF‐like cells derived from stimulation with SKOV3‐CM increased the invasive ability of the heterogeneous spheroids on Day 4 of culture (Figure 7C). Overall, heterogeneous spheroids demonstrated a greater ability to invade the surrounding matrix than the homogeneous control spheroids. Irrespective of the cell line's specific differences, CAF‐like cells had a significant impact on tumor invasion.

## 3 Discussion

In this work, we generated heterogeneous CAF‐like cells by exposing human dermal fibroblasts to tumor‐derived stimuli. The CAF‐like cells generated were then incorporated into a complex 3D OC model to simulate heterogeneous clusters of cells, resembling that of ascitic fluid of OC patients.

Previous studies have generated CAF‐like phenotypes by culturing stromal cells with tumor‐CM or exposing them to exosomes [52, 53, 54, 55]. However, these studies primarily focus on specific components derived from tumor cells, such as exosomes or EVs, and primarily generate a single subtype of CAF, often neglecting the complex interplay of various tumor‐secreted factors and the diverse heterogeneity of CAF. In contrast, our work demonstrates that different OC cell lines produce heterogeneous CAF‐like populations, influenced by the genetic background of tumor cells and the presence of TGF‐β.

CAF‐like cells generated in this study, as described in the literature, were characterized based on changes in cytoskeletal architecture, particularly the expression of αSMA [56, 57], migration [58], and the expression of key markers such as αSMA, FAP, PDPN, IL‐6, and TGF‐β [59]. Notably, OV‐90‐CM and conditions with TGF‐β led to increased αSMA^+^ cells, accompanied by structural alterations typical of myofibroblasts involved in wound healing [60]. On the other hand, αSMA diffuse signal in the cytoplasm of quiescent fibroblasts was reported [61]. Additionally, PDPN^+^ cells increased in response to HEY‐CM and SKOV‐CM, alone or with TGF‐β, along with upregulated gene expression of αSMA and PDPN in the same conditions.

Further phenotypic characterization revealed that FAP, a fibroblast activation broad marker [62], was upregulated in all OC‐CM conditions, regardless of TGF‐β, while MFAP5 was selectively upregulated by OV‐90‐CM, SKOV3‐CM, and TGF‐β. MFAP5 is of interest for its role in collagen deposition [39] and integrin signaling [32], contributing to CAF‐mediated pro‐invasive functions [63]. CAF‐like cells also showed increased deposition [64] and, in particular for OV‐90‐CM treatment, COL11A1 secretion [48], consistent with a CAF‐related ECM remodeling role. We further evaluated fibroblast activation by assessing their migratory profile [65] and secretion of IL‐6 [66]. The migration capacity has been reported for CAF involved in tumor cell metastasis [67]. Migration assays indicated that OC‐CM alone enhanced fibroblast motility, though not significantly. The addition of TGF‐β further increased migration, emphasizing its functional impact on fibroblast activation. IL‐6 production characterizes the iCAF subtype [68], contributing to immune modulation. All OC‐CM treatments increased IL‐6 secretion compared to controls [69, 70], suggesting that IL‐6 secretion is primarily driven by tumor‐derived factors. Interestingly, the degree of IL‐6 secretion varied among cancer cell lines, with HEY‐CM inducing the highest levels, followed by OV‐90‐CM and SKOV3‐CM. Notably, HEY‐CM and SKOV3‐CM contain IL‐6 (data not shown), while OV‐90‐CM contained higher VEGF [71, 72] and COL11A1 levels when compared to HEY‐CM and SKOV3‐CM (data not shown), reflecting the varying composition of each OC‐CM as a consequence of their genomic differences [73]. These differences likely drive the preferential differentiation of fibroblasts into distinct CAF subtypes, highlighting the influence of tumor‐derived factors on CAF heterogeneity [74].

Gene expression analysis revealed distinct profiles depending on the cancer cell line and the presence of TGF‐β. HEY‐CM and SKOV3‐CM elicited similar effects, while OV‐90‐CM and OV‐90‐CM/TGF‐β upregulated a unique set of genes. Across all conditions, TGF‐β led to downregulation of the genes encoding CD26, FSP‐1, CXCL12, CD74, and CD39, underscoring its role in modulating specific fibroblast pathways.

Single‐cell sequencing analysis of OC has previously highlighted the heterogeneity of CAF within the TME [75]. In this study, we propose that our experimental setup fosters an environment capable of generating diverse CAF‐like subpopulations, corresponding to either myCAF or iCAF characteristics [46, 47, 48]. HEY‐CM and SKOV3‐CM were mainly associated with the induction of iCAF‐like phenotypes, characterized by increased PDPN^+^ cells, IL‐6 secretion, and upregulated expression of *CXCL2*, *CXCL8*, and *MMP1*. Importantly, we observed that HEY‐CM also induced the secretion of IL‐1β, suggesting a pro‐inflammatory shift in fibroblast activation. Conversely, OV‐90‐CM, particularly when in association with TGF‐β, stimulates myCAF‐like phenotypes characterized by αSMA expression and COL11A1 secretion. The variations related to OC‐CM specification highlight the need for further analysis to identify the specific factors responsible for these effects and to investigate the heterogeneity of the CAFs generated. Although this work lacks a direct comparison with patient‐derived CAF, the combination of OC‐CM with TGF‐β presents significant potential. This approach not only offers a straightforward method for generating heterogeneous CAF‐like cells in the absence of CAF obtained from OC biopsies, but it also provides a valid experimental framework to dissect the complex interactions that occur between OC cells or their secreted factors and CAFs. By mimicking the TME through OC‐CM and TGF‐β treatment, our setup enables the study of the cross talk, signaling pathways, and functional roles that govern the formation, behavior, and diversity of CAF‐like populations, offering insights that could enhance our understanding of CAF formation in the tumor milieu.

To further explore CAF‐tumor interactions, we generated a 3D heterogeneous spheroid model comprising tumor and CAF‐like cells. Fibroblasts were predominantly localized in the core of spheroids, while tumor cells concentrated at the periphery, mimicking the structure of ascitic spheroids observed in patients [49]. Spheroids formed with HEY cells alone or with fibroblasts showed similar growth patterns, whereas OV‐90 and SKOV3 spheroids exhibited enhanced growth and compaction when fibroblasts were included [78]. These findings indicate that CAF‐like cells support tumor growth and structural integrity, consistent with their roles in contractility and ECM deposition.

The material exchange between CAF‐like cells and tumor cells was evident in our model, with increased transfer observed in HEY‐CM and SKOV3‐CM‐derived CAF‐like cells, particularly in the presence of TGF‐β. This intercellular communication can enhance the reciprocal signaling that fosters a tumor‐supportive microenvironment, thereby promoting cancer cell survival, invasive behavior, and resistance to therapeutic treatments [70, 79]. Moreover, OC‐CM treatment reduced CD90 expression in CAF‐like cells within heterogeneous spheroids. Low CD90 expression in mesenchymal stromal cells has been linked to increased IL‐6 secretion, EMT induction, and chemoresistance in glioblastoma [80], as well as contributing to tumor vascularization and immunosuppression due to their higher production of VEGF and PGE2 [81]. In OC, CD90 variability among CAF subtypes suggests its potential role in defining functional CAF populations. Our data indicate that OC‐CM drives the generation of CAF‐like cells with distinct CD90 expression profiles, potentially contributing to tumor‐promoting functions.

To assess the impact of CAF‐like cells on tumor invasiveness, we examined spheroid invasion in the ECM‐mimicking matrix [82]. HEY spheroids with CAF‐like cells derived from HEY‐CM or HEY‐CM/TGF‐β exhibited increased invasion compared to homogeneous spheroids. Similarly, OV‐90 and SKOV3 spheroids acquired invasive capacity in the presence of CAF‐like cells. These results underscore the role of CAF‐like cells in enhancing the invasive potential of OC spheroids.

In summary, our work presents an experimental setup capable of inducing CAF‐like cells with different characteristics by exploiting the secreted factors and cell derivatives contained in OC‐CM. This approach captures CAF diversity and provides an in vitro model where variables (i.e., the ratio between cancer cells and fibroblasts, days of culture, external stimuli) can be controlled, maintaining a high reproducibility of experiments. The model is well‐suited for high‐throughput drug screening and mechanistic studies, offering an alternative to patient‐derived samples and reducing reliance on in vivo models. Nevertheless, we acknowledge the limitation of our study in not including direct experimental evaluations as in vivo settings of the functional interactions between stimulated fibroblasts and tumor cells. Although there are still open questions about the factors involved in the OC‐CM effect on fibroblasts and the interaction that takes place inside TME, our work might provide a reliable tool to study these mechanisms between stromal and tumor cells in order to find a new therapeutic tool to contrast OC development and dissemination.

## 4 Materials and Methods

### 4.1 Isolation and Culture of Fibroblasts

Dermal fibroblasts were isolated from skin biopsies derived from the foreskin of healthy patients (*n* = 5) after informed consent, in accordance with the guidelines established by the local ethics committee “Comitato Etico Provinciale di Brescia,” Italy (number NP 2296, April 4, 2023). Skin fragments were soaked in sterile saline solution (NaCl 0.9%, Fresenius Kabi, Bad Homburg vor der Höhe, Germany) added with 0.25% povidone‐iodine (B. Braun Melsungen AG D‐34209 Melsungen, Germany) for 2 s and incubated for 3 min with phosphate‐buffered saline solution (PBS, Euroclone, Milano, Italy) + Penicillin/Streptomycin (P/S) (Euroclone) + Amphotericin B (Euroclone) + Cefamezin (TEVA). Finally, two additional washes were performed with PBS. The biopsies were then cut into smaller pieces using a sterile scalpel on a petri dish. The fragments were placed in six‐well plates (5–10 fragments/well) and cultured in DMEM (Euroclone; ECM0749L) with 20% heat‐inactivated fetal bovine serum (FBS) (Euroclone; ECS5000LH), 2 mM L‐glutamine (Euroclone; ECB3000D), and 1% P/S. Cultures were monitored for fibroblast adhesion to plastic. Once colonies formed, skin fragments were removed, and the culture medium was replaced with the fresh medium until fibroblasts reached 80%–90% confluency. Then, cells were expanded until Passages 3 and 4 in DMEM 20% FBS. For subsequent passages and for experimental conditions, fibroblasts were cultured in DMEM 10% FBS.

### 4.2 OC Cell Lines and Generation of Conditioned Medium From the Culture of HEY, OV‐90, and SKOV3 Cells

HEY and OV‐90 human OC cell lines were kindly provided by Daniela Gallo (Fondazione Policlinico Universitario “Agostino Gemelli” IRCCS, Rome, Italy); SKOV3 was obtained from the American Type Culture Collection (ATCC). HEY cells were cultured in RPMI 1640 (Euroclone; ECB9006LX10) supplemented with 10% FBS and 1% nonessential amino acids (Thermo Fisher; 11140‐035). OV‐90 cells were cultured in a complex medium composed of MCDB (Sigma‐Aldrich, St. Louis, Missouri, USA; M‐6770) plus M199 (Merk; M4530) at a 1:1 ratio and 0.5% MEM (Sigma‐Aldrich; 56416C‐1L), supplemented with 15% FBS. SKOV3 cells were cultured in McCoy's medium (Euroclone, ECM0210L) supplemented with 10% FBS. All culture media were supplemented with 2 mM L‐glutamine and 1% P/S.

OC‐CM was generated from three different OC cell lines: HEY cells, derived from a human OC xenograft originally grown from a peritoneal deposit of a patient with moderately differentiated papillary cystadenocarcinoma [83]; OV‐90 cells, originally isolated from malignant ascites from patients with ovarian adenocarcinoma, harboring p53 mutations and exhibiting genomic features similar to HGSOC; and SKOV3 cells, derived from the ascites of a patient with ovarian adenocarcinoma and lacking the expression of p53 at the protein level.

To obtain conditioned medium derived from HEY (HEY‐CM), OV‐90 (OV‐90‐CM), and SKOV3 (SKOV3‐CM) OC cell lines, cells were seeded in T182 flasks at 10,000 cells/cm^2^ (HEY, SKOV‐3) and 20,000 cells/cm^2^ (OV‐90). Upon reaching 80% confluency, the cell culture medium was removed and replaced with serum‐free DMEM (2 mM L‐glutamine, 1% P/S). The medium was collected after 48 h of incubation, centrifuged at 300 × *g* for 10 min to remove dead cells and debris, and filtered through a 0.2‐µm sterile filter. OC‐CMs were stored at −80°C until use.

### 4.3 Induction of CAF‐Like Cells

To obtain CAF‐like cells, dermal fibroblasts were seeded in T25 flasks at 10,000 cells/cm^2^. Fibroblasts were cultured in different conditions: (a) DMEM; (b) DMEM + TGF‐β; (c) HEY‐CM; (d) HEY‐CM + TGF‐β; (e) OV‐90‐CM; (f) OV‐90‐CM + TGF‐β; (g) SKOV‐3‐CM; (h) SKOV3‐CM + TGF‐β. Culture media for each treatment were composed of 75% OC‐CM (supplemented with 10% FBS) and 25% DMEM (supplemented with 10% FBS). TGF‐β (Miltenyi; 130‐095‐067) was used at a final concentration of 10 ng/mL. Cells were collected 3 and 7 days after culture and subjected to further analyses.

To monitor phenotype stability, fibroblasts that were differentially treated were frozen, thawed, and cultured in FBS 10% DMSO (AppliChem, APA36720100) for 3 days. Alternatively, they were detached with trypsin, reseeded in DMEM 10% FBS, and cultured until P2.

### 4.4 Generation of Homogeneous and Heterogeneous Spheroids

Spheroids were generated using a total of 6000 cells. For homogeneous spheroids, 6000 HEY, OV‐90, or SKOV‐3 cells were used. For heterogeneous spheroids, a 1:1 ratio of tumor cells (3000) and fibroblasts (3000), previously subjected to different pretreatments (as described in “Induction of CAF‐like cells”), were used. After detachment, the cells were counted and combined to obtain spheroids with the following conditions: (1) tumor cells; (2) tumor cells and normal fibroblasts cultured in DMEM 10% FBS; (3) tumor cells and fibroblasts cultured in DMEM 10% FBS + TGF‐β; (4) tumor cells and fibroblasts cultured in OC‐CM; (5) tumor cells and fibroblasts cultured in OC‐CM + TGF‐β. To allow spheroid formation, 2% methylcellulose (Sigma‐Aldrich; M7027) was added to the cell resuspension medium (20% of total volume). The cells were then seeded in U‐bottom 96‐well plates (Corning; 3788) in a final volume of 200 µL/well and kept in culture until Day 6.

To calculate spheroid growth, six to eight images/condition of the spheroids were taken using an Olympus 1 × 50 microscope equipped with an OPTICA camera (4083.13), at 4× magnification, at Days 1, 3, and 6. The areas of spheroids were measured with ImageJ and reported in pixels [2].

ImageJ calculations: Spheroid area was calculated using the “Macros” plugin in ImageJ. Images were converted to grayscale. Images less than 1000 pixels [2] were excluded, and any holes in spheroids were automatically filled. The area of the resulting image was obtained automatically in ImageJ [84].

### 4.5 Cell Labeling and Microscopic Analysis of Cell Distribution

Treated fibroblasts and tumor cells were labeled by incubating cells with 2 µM red CMTPX (Thermo Fisher, C34552) or 5 µM green CMFDA (Thermo Fisher, C7025), respectively. Cells were used to form spheroids, as described above. Spheroid images were acquired using a Nikon Eclipse Ni‐U microscope equipped with a Mono Camera Nikon DS‐Fi3 Version 4.60.

### 4.6 Spheroid Invasion Assay

Homogeneous and heterogeneous spheroids of HEY and SKOV‐3 were harvested in 50 µL and transferred into a 96‐well flat plate pre‐coated with 50 µL of Geltrex (Thermo Fisher; 955922). OV‐90 spheroids were harvested, mixed with 70 µL of Geltrex, and transferred into a 96‐well flat plate. A total of 100 µL of DMEM 10% FBS was added to each well. Images of the spheroids were acquired on Days 1, 2, and 4 after transfer using an Olympus 1 × 50 microscope equipped with an OPTICA camera (4083.13) at 4× magnification, and the invasive area was calculated with ImageJ by subtracting the spheroid core area from the total invaded area.

ImageJ calculations: Pictures of a spheroid on Geltrex‐coated wells were analyzed as described above. The total area of the spheroids (A) was measured manually, considering all sprouts that could be observed. Afterward, the area of the solid spheroids (B) (from which sprouts originate) was also measured. Data were then exported, and final values reported as invasive area (C) were obtained as follows: C = A − B.

### 4.7 Spheroid Disaggregation and Staining

One day after seeding, heterogeneous spheroids with CMTXP‐labeled fibroblasts were harvested in 50 µL of culture medium and transferred to a 48 well/plate. Excess medium was discarded, and 300 µL of trypsin was added to each well. Plates were then incubated for 5 min at 37°C and vigorously resuspended afterwards for 15–20 times. Once disaggregation was verified with microscopy, 300 µL of FBS was added, and samples were harvested for the staining procedure. Dead cells were excluded using the eBioscience Fixable Viability Dye eFluor 780 (Thermo Fisher Scientific) according to the manufacturer's instructions, and then the cells were stained for 20 min at 4°C with the CD90 BUV563 antibody (BD, 741382). Samples were acquired on FACS Symphony A3 (BD Bioscience). Data were analyzed with FlowJo 10.8. After the exclusion of dead cells, fibroblast CMTXP‐positive cells were analyzed for the different expressions of CD90 (negative, low, high).

Quantitative real‐time PCR, flow cytometry analysis, immunofluorescence, wound healing, transwell migration, IL‐6 and IL‐1β quantification, and western blot methods are in the Supporting Information.

### 4.8 Statistical Analysis

Data report the mean and standard deviation (in histograms, individual values also are reported). The parameters were compared using a two‐way analysis of variance after logarithmic transformation of data (ANOVA), with Bonferroni or Dunnett multiple comparison tests post‐analysis. Data are representative of three to five experiments (*n* is reported in each figure legend). For the CAF‐related gene expression analysis, generalized linear mixed models (with a log link function for gamma‐distributed data) were performed with a random effect on dermal fibroblasts and gene (15 different genes), CM (8 CM, with one as reference DMEM), and their interaction (gene × CM) as a fixed effect repeated within dermal fibroblasts.

## Author Contributions

J.R. and P.C. conceived the experiments, J.R., P.C., P.B.S., E.V., E.G., and A.P. conducted the experiments, P.C., J.R., P.B.S., and E.V. analyzed the results. F.C. performed statistical analysis on gene expression. J.R., P.C., A.P., F.R.S., A.S., and O.P. wrote or provided input toward the manuscript. All authors have read and approved the manuscript.

## Ethics Statement

Samples were collected after informed consent, in accordance with the guidelines established by the local ethics committee “Comitato Etico Provinciale di Brescia,” Italy (number NP 2296, April 4, 2023).

## Conflicts of Interest

The authors declare no conflicts of interest.

## Supporting information



Supporting information

## Data Availability

The data that support this study are available from the corresponding author upon reasonable request.
