# Supplementary material for: Modeling Stromal Cells Inside the Tumor Microenvironment of Ovarian Cancer: In Vitro Generation of Cancer‐Associated Fibroblast‐Like Cells and Their Impact in a 3D Model
Source: MedComm (2020). 2025 Apr 17;6(5):e70172. doi: 10.1002/mco2.70172 (PMC12006666; doi:10.1002/mco2.70172)
Supplement: Supplementary file 1 — Supporting information [file MCO2-6-e70172-s001.docx]

Supporting Information for:

**"Modeling stromal cells inside the tumor microenvironment of ovarian cancer: in vitro generation of cancer associated fibroblast-like cells and their impact in a 3D model"**

Running title: Modeling stromal cells in ovarian cancer TME

Jacopo Romoli 1*, Paola Chiodelli 1*, Patrizia Bonassi Signoroni 2, Elsa Vertua 2, Clarissa Ferrari 3, Elisabetta Giuzzi 2, Alice Paini 2, Elisa Scalvini 2, Andrea Papait 1,4, Francesca Romana Stefani 2, Antonietta Rosa Silini 2 and Ornella Parolini 1,4#.

1 Department of Life Science and Public Health, Università Cattolica del Sacro Cuore, 00168 Rome, Italy.

2 Centro di Ricerca E. Menni, Fondazione Poliambulanza Istituto Ospedaliero, 25124 Brescia, Italy.

3 Research and Clinical Trials Unit, Fondazione Poliambulanza Istituto Ospedaliero, 25124 Brescia, Italy.

4 Fondazione Policlinico Universitario “Agostino Gemelli” IRCCS, 00168 Rome, Italy.

*Contributed equally to this work.

# Corresponding author: Email: [ornella.parolini@unicatt.it](mailto:ornella.parolini@unicatt.it)

***
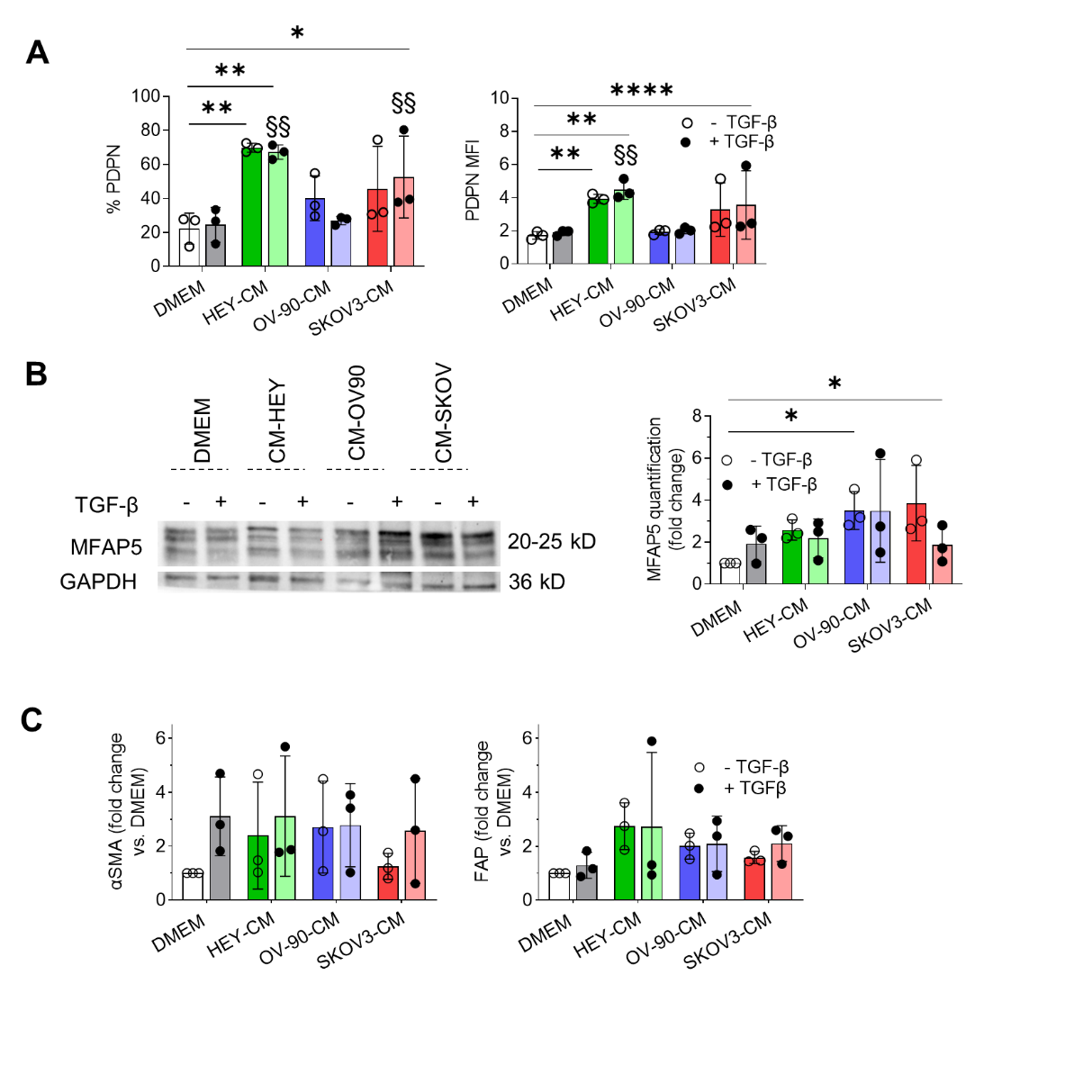
***

***Figure S1 – Ovarian cancer cell conditioned media and TGF-β induce CAF-related marker expression***

*A) Dermal fibroblasts were exposed to HEY-CM, OV-90-CM and SKOV3-CM in the absence or presence of TGF-β for 3 days. PDPN markers were evaluated by flow cytometry. The results show percentage of PDPN^+^ cells and the relative mean fluorescence intensity (MFI); n=3. B) Cell lysates were analysed by western blot with MFAP5 antibody and normalized for GAPDH. Molecular weights of the respective bands, expressed in kDa, are shown on the right. The right panel shows densitometric analysis of immunoreactive bands. The normalization was made on 20-25 kDa bands. C) Densitometric analysis of immunoreactive bands in presented in figure 1F. αSMA and FAP normalization performed on 42 kDa and 90 kDa bands respectively.*

** p < 0.05; ** p < 0.01; **** p<0.0001; treatments vs. – TGF-β. §§ p < 0.01; treatments vs. DMEM + TFG-β.*

*
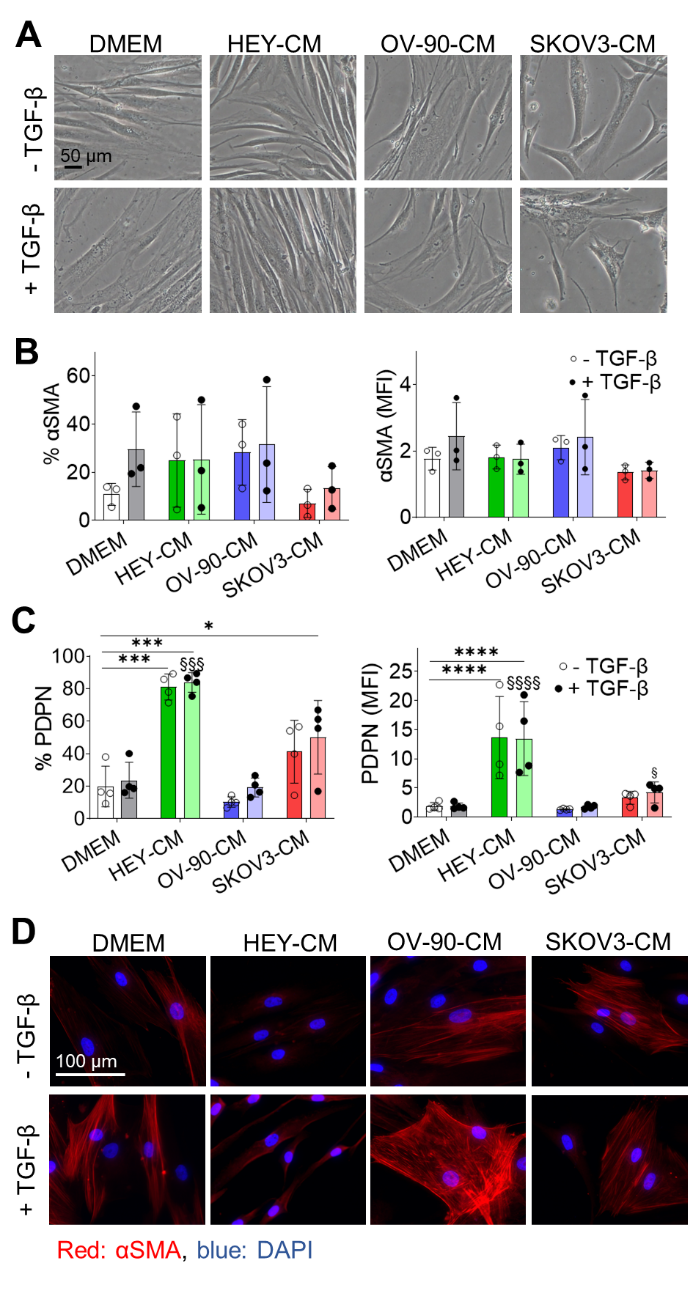
*

***Figure S2 –*** ***Analysis of αSMA and PDPN expression on adult dermal fibroblasts.***

*Adult dermal fibroblasts were exposed to HEY-CM, OV-90-CM and SKOV3-CM in absence or presence of TGF-β for 7 days. A) Representative images of adult dermal fibroblasts cultured under different conditions for 7 days. Scale bar = 50 μm. B) αSMA and PDPN protein expression evaluated by flow cytometry. The results show percentage of αSMA^+^ and PDPN^+^ cells and the relative mean fluorescence intensity (MFI). n=3-4. C) Cells were stained with αSMA antibody (red), nuclei were counterstained with DAPI (blue). Pictures were acquired at 63x magnification (C, scale bar 100 μm). n=4. * p < 0.05; *** p< 0.001; **** p<0.0001 treatments vs. – TGF-β. §§§ p < 0.001; §§§§ p < 0.0001 treatments vs. DMEM + TFG-β.*


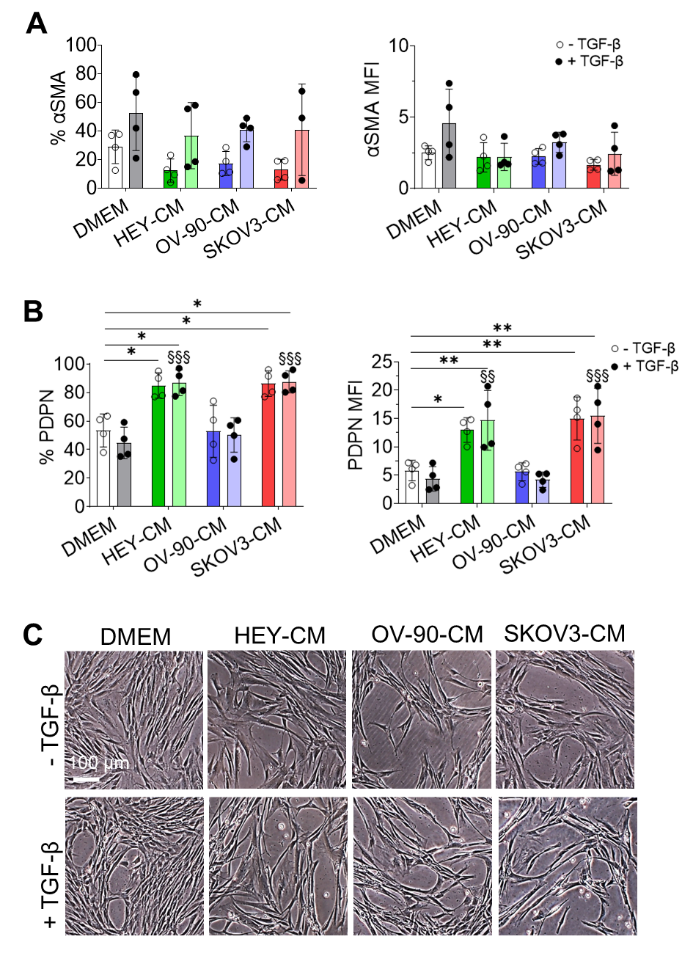


***Figure S3– CAF-like cell markers and morphology after one cycle of freeze/thaw***

*Dermal fibroblasts were exposed to HEY-CM, OV-90-CM and SKOV3-CM in the absence or presence of TGF-β for 7 days. The cells were cultured without CM after one freeze/thaw (F/T) cycle, then PDPN (A) and αSMA (B) markers were evaluated using flow cytometry. The percentage of αSMA^+^ and PDPN^+^ cells and the relative mean fluorescence intensity (MFI) were determined. n=3-4. * p < 0.05; ** p < 0.01; treatments vs. – TGF-β; §§ p < 0.01, §§§ p < 0.001; treatments vs. DMEM + TFG-β. C) Representative images of fibroblasts cultured under reported conditions and treatments. Scale bar 100 μm.*

***
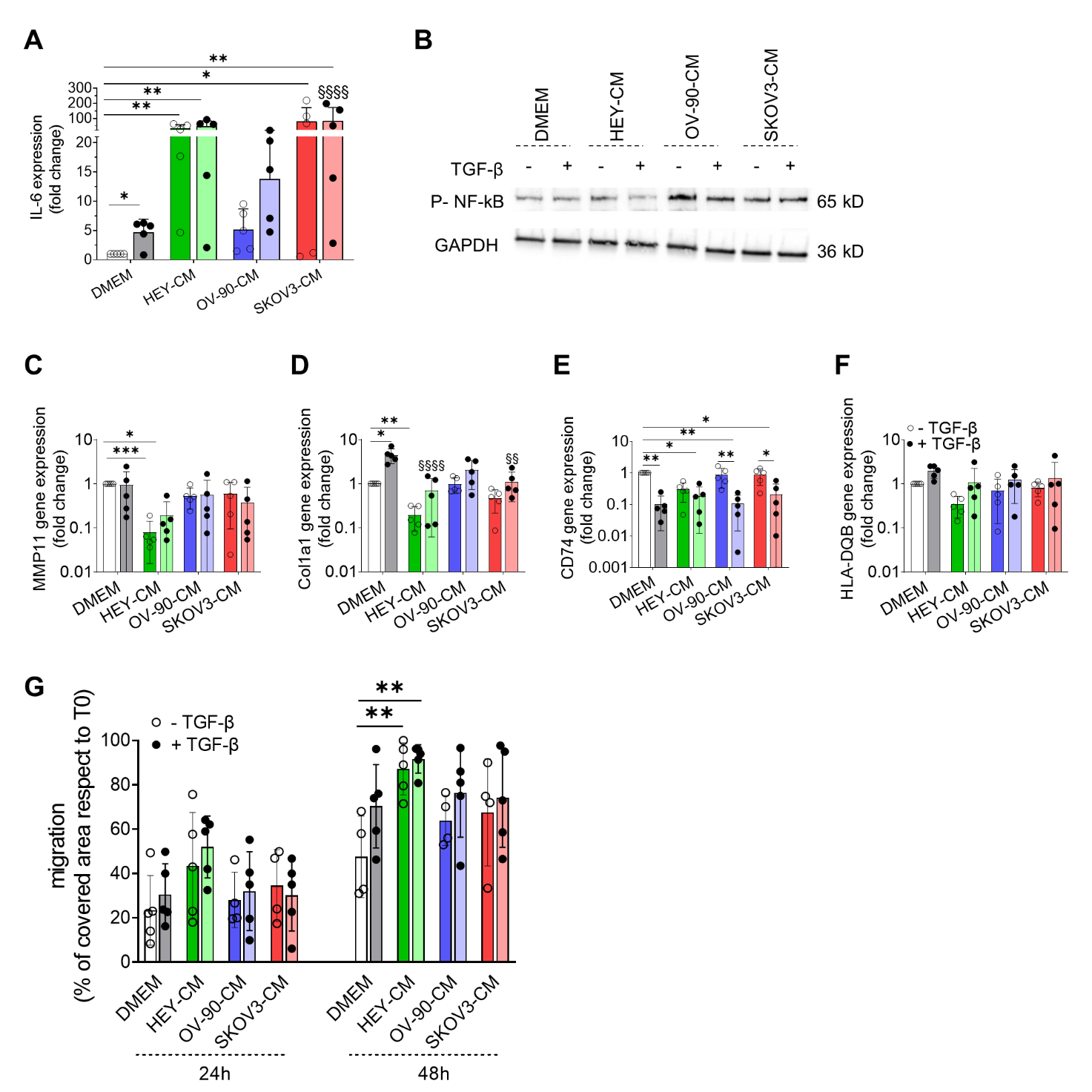
***

***Figure S4 – Analysis of CAF-like acquired secretory and migration functions***

*Dermal fibroblasts were exposed to HEY-CM, OV-90-CM and SKOV3-CM in the absence or presence of TGF-β for 7 days. A) IL-6 expression was analyzed by RT-PCR. The changes of gene expression were expressed as fold-change relative to DMEM treatment, used as control. n=5. B) Cell lysates were analysed by western blot with p-NF-kB antibodies and normalized for GAPDH. Molecular weights of the respective bands, expressed in kDa, are shown on the right. C-F) MMP11, COL1A1, CD74 and HLA-DQB expression was analyzed by RT-PCR changes in gene expression are presented as fold-change relative to DMEM treatment, used as control, in a logarithmic scale. n=5. G) After the above-mentioned treatments, cells were detached and seeded in ibidi chambers for wound healing assay. The wound closure was monitored for 48h. Data are reported as the percentage of the covered area compared to T0 after 24 and 48 hours. n=3-4. * p < 0.05; ** p < 0.01; *** p<0.001; treatments vs. – TGF-β. §§ p<0.01; §§§§ p<0.0001 treatments vs. DMEM + TFG-β.*

***
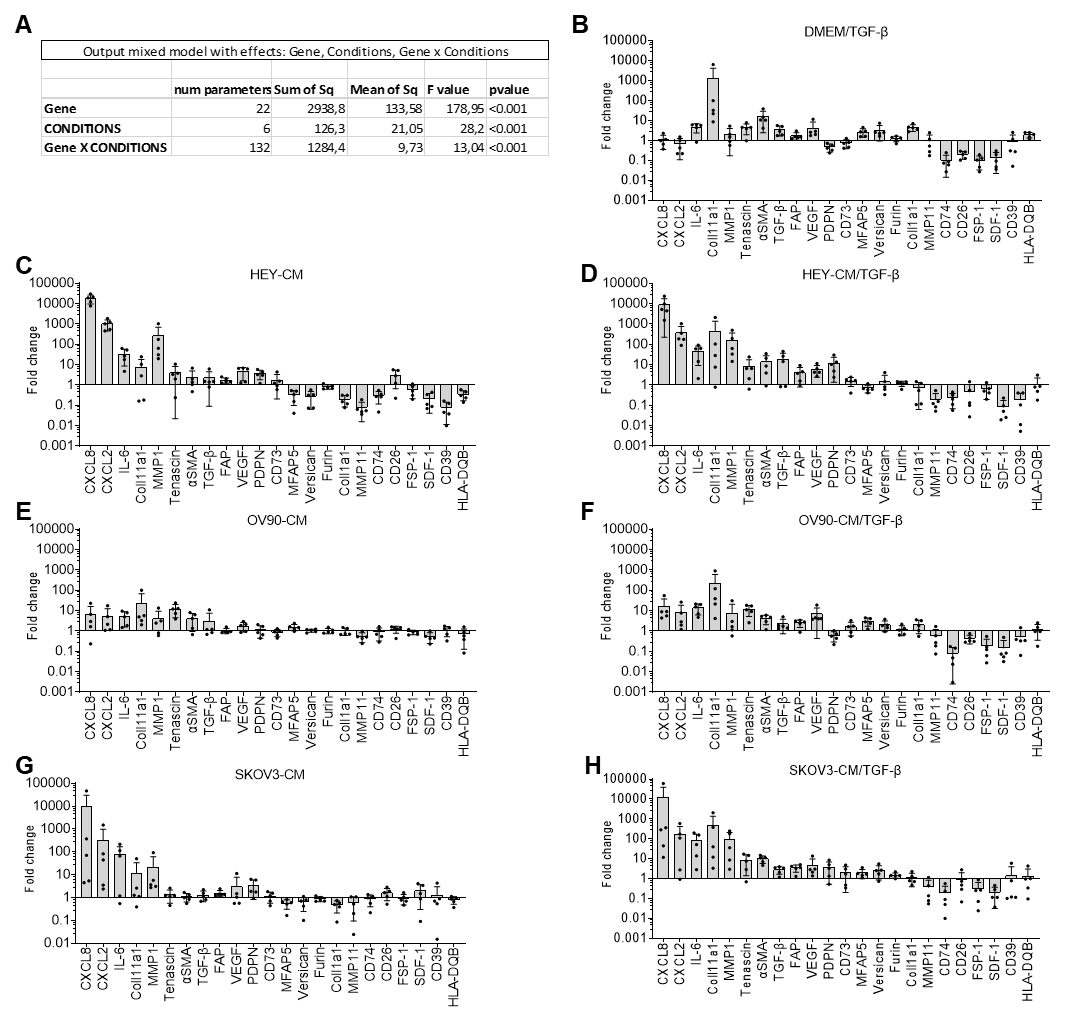
 Figure S5 – Gene expression profile of fibroblasts 7 days after treatment with HEY-CM, OV-90-CM and SKOV3-CM in the absence or presence of TGF-β for 7 days.***

*A) Generalized linear mixed model output: parameter estimation and p values of the fixed effects: gene, conditions and the interaction gene x conditions. B-H) Gene expression profile in DMEM/TGF-β (B), HEY-CM (C), HEY-CM+TGF-β(D), OV90-CM(E); OV90-CM+TGF-β(F), SKOV3-CM(G) and SKOV3-CM+TGF-β(H) conditions. y-axes are expressed as logarithmic scale.*

**Supplementary Material and methods:**

**Quantitative Real-Time PCR**

To evaluate the expression of fibroblast-associated genes, cells were collected in RLT buffer (Qiagen, Frederick, MD, USA) 7 days after treatment and stored at -80°C until use. Once thawed, total RNA was extracted using the EZ1 RNA cell Mini Kit (Qiagen), in a BioRobot EZ1 Advanced XL Workstation. The iScript Advanced cDNA Synthesis Kit for RTqPCR (Biorad, Hercules, California, USA) was used for cDNA synthesis. cDNA was pre-amplified with SsoAdvanced PreAmp Supermix (Biorad). Real-time PCR was performed using the Biorad instrument CFX96 Quantitative Real-Time PCR. The real-time PCR cycling program was as follows: 30 seconds at 95°C, 40 cycles of 10 seconds at 95 °C, 20 seconds at 58 °C. See Supplementary Information for primer sequences (Table S1). Data were analyzed with Biorad CFX Maestro 2.2 (Biorad). The clustering was obtained by using “MORFEUS” online tool, which shows the links between the condition considering the pattern of up-regulated or down-regulated genes in the different conditions (One minus Pearson correlation).

***Table S1.*** *Primer sequences (Sigma Aldrich) of genes analyzed by quantitative real-time PCR.*

| **Protein (reported in figure)** | **Gene name** | **Forward** | **Reverse** |
| --- | --- | --- | --- |
| **αSMA** | *ACTA2* | TGGCTATTCCTTCGTTACTACTGCT | CATCAGGCAACTCGTAACTCTTCTC |
| **CD26** | *DPP4* | ATGCCAGGAGGAAGGAATCT | TTCCAGGACTCTCAGCCCTTT |
| **CD39** | *ENTPD1* | CTGATTCCTGGGAGCACAT | GACATAGGTGGAGTGGGAGAG |
| **CD73** | *NT5E* | GCCTGGGAGCTTACGATTTTG | TAGTGCCCTGGTACTGGTCG |
| **CD74** | *CD74* | AAGCCTGTGAGCAAGATGCGCA | AGCAGGTGCATCACATGGTCCT |
| **Col1a1** | *COL1A1* | GATTCCCTGGACCTAAAGGTGC | AGCCTCTCCATCTTTGCCAGCA |
| **Col11A1** | *COL11A1* | TGGTGATCAGAATCAGAAGTTCG | AGGAGAGTTGAGAATTGGGAATC |
| **CXCL2** | *CXCL2* | GGCAGAAAGCTTGTCTCAACCC | CTCCTTCAGGAACAGCCACCAA |
| **CXCL8** | *CXCL8* | GAGAGTGATTGAGAGTGGACCAC | CACAACCCTCTGCACCCAGTTT |
| **CXCL12** | *CXCL12* | AGCCAACGTCAAGCATCTCA | GTCCTCATGGTTAAGGCCCC |
| **FAP** | *FAP* | GAAAGAAAGGTGCCAATA | GATCAGTGCGTCCATCA |
| **FSP-1** | *S100A1* | TCTGAGATGTGGGCTTGCAC | GGACACCATCACATCCAGGG |
| **Furin** | *FURIN* | GCAAAGCGACGGACTAAACG | TGCCATCGTCCAGAATGGAGA |
| **HLA-DQB** | *HLA-DQB1* | GAGCAAGATGCTGAGTGGCGTT | GTCTCAGGAGTCAGTGCAGAAG |
| **IL-6** | *IL6* | AGTCCTGATCCAGTTCCTGC | CTGGCATTTGTGGTTGGGTC |
| **MFAP5** | *MFAP5* | GCCAGCCAAAGTAGGAACAG | AGCAAGAAACAGCAGCACCT |
| **MMP1** | *MMP1* | GACCTGGAGGAAATCTTGC | GTTAGCTTACTGTCACACGC |
| **MMP11** | *MMP11* | GAGAAGACGGACCTCACCTACA | CTCAGTAAAGGTGAGTGGCGTC |
| **PDPN** | *PDPN* | CGAAGATGATGTGGTGACTC | CGATGCGAATGCCTGTTAC |
| **Tenascin** | *TNC* | TCTCTGCACATAGTGAAAAA | TCAAGGCAGTGGTGTCTGTGA |
| **TGF-β1** |  | GCGTGCTAATGGTGGAAACC | GAGCAACACGGGTTCAGGTA |
| **VEGF** |  | TTGCCTTGCTGCTCTACCTCCA | GATGGCAGTAGCTGCGCTGATA |
| **Versican** |  | GCACCTGTGTGCCAGGATA | CAGGGATTAGAGTGACATTCATCA |

**Flow Cytometry Analysis**

Dermal fibroblasts were collected 3 and 7 days after culture. Dead cells were excluded using the eBioscienceTM Fixable Viability Dye eFluorTM 780 (Thermo Fisher Scientific) according to the manufacturer’s instructions. Cells were subsequently stained for 20 minutes at 4°C with PDPN BUV395 antibody (BD Bioscence; 747630). After membrane staining, intracellular staining with αSMA Alexa Fluor 488 (Life Technologies, 53-9760-82) was performed after fixation and permeabilization using BD Cytofix/Cytoperm (BD Biosciences) 25 minutes at room temperature (RT) in the dark. Samples were acquired on FACS Symphony A3 BD (BD Bioscience). Data were analyzed with FlowJo 10.8.

**Immunofluorescence**

Fibroblasts were seeded at 10.000 cells/cm^2^ on round glass supports placed inside 24 well plates. After 7 days of treatment, cells were fixed in 4% formalin (DIAPATH, F0043) for 2h at RT. Normal goat serum (Invitrogen; 10000 C) was then used 1:10 in TBST 1X to block unspecific binding, 30 minutes in the dark at RT (Reagents for TBST: SDS (Sigma Aldrich; 654833), Glycin (Merck; G8898), Tris (M-Medical, IB70144). Primary antibodies for αSMA (Dako, M0851) and MAGP-2/MFAP5 (Novus, NBP1-82803) were used 1:100 and 1:500 (in TBST) respectively, and incubated overnight (4°C, in the dark). Secondary antibodies horse anti-mouse Dylight 488 (Vector, DI-2488 for αSMA) and Vector, goat anti-rabbit Dylight 594 (Vector, DI-2488, for MFAP5), were added at the concentration of 5 µg/ml and incubated for 90 minutes. Slides were prepared using Vectashield vibrance antifade mounting medium with DAPI (Vector, H-1800). Images were acquired using a Nikon Eclipse Ni-U microscope equipped with Mono Camera Nikon DS-Fi3 Version 4.60 at 20× and 40× magnification or MICA microscope (Leica Microsystem) at 63x magnification.

**Wound healing assay**

Dermal fibroblasts were detached 7 days after treatment and seeded in silicon inserts (ibidi GmbH. Gräfelfing, Germany; 81176) in 24 well plates. Two inserts were used for each condition, in each experiment performed. Cells were seeded at density of 60.000/cm^2^. The day after, inserts were removed and pictures were acquired at day 0 (T0), day 1 and day 2 using Olympus 1X50 microscope, equipped with OPTIKA camera (4083.13), using 4× magnification. The extent of wound repair was evaluated by measuring the area of the wound by computerized image analysis (area at day 1 or day 2 in respect to T0) using the ImageJ software (http://rsb.info.nih.gov/ij/).

**Transwell migration assay**

Dermal fibroblasts were detached 7 days after treatment and seeded in 8 μm transwell supports (Corning, Glendale, Arizona, USA; CL-β422-48EA), placed in 24 well plates. Cells were seeded at density of 80,000 cells/insert, in a volume of 150 µl of serum free DMEM. The insert was then placed into a 24 well plate containing 500 µl of DMEM 20% FBS. After 48 hours, transwells were removed and the upper part of the inserts was cleaned using swabs to remove cells that did not migrate. Cells on the lower part of the supports were then fixed in ice cold methanol for 15 minutes and left to dry. Afterwards, crystal violet (Sigma Aldrich; V5265) staining was performed by soaking inserts for 10 minutes and the upper part was cleaned again with swabs. Once inserts were dry, 3 images/insert were acquired using Olympus 1X50 microscope, equipped with OPTIKA camera (4083.13), using 4× magnification, and the number of stained cells was counted manually. Crystal violet was then solubilized in 200 µl of 33% acetic acid (Carlo Erba; 2789). Finally, 160 µl of the solubilized solution was transferred into a 96 well plate and analyzed with VictorTM X4 (Perkin Elmer, Waltham, Massachusetts, USA) at 595 nm.

**IL-6, IL-1β and Col11a1 quantification**

Supernatants derived from fibroblast treatment were collected, centrifuged at 300g to remove cells and debris and stored at -80°C. IL-6 ELISA kit (Invitrogen, Waltham, Massachusetts, USA; 88-7066) was used following manufacturer’s instructions. Briefly, 96 well flat-bottom plates were coated with 100 μL/well of capture antibody and incubated overnight at 4°C. The day after, washes were performed and blocking solution was added. Samples (diluted 1:1000) and standards were pipetted (100 μL/well) to the appropriate wells and incubated at 4°C overnight. IL-6 detection antibody was added and incubated for 1 hour at RT. After washes, streptavidin-HRP was added in each well and incubated for 30 minutes at RT, then 100 µl/well of (3,3',5,5'-tetrametilbenzidina) TMB were added for 15 minutes at RT. Finally, 100 μL/well of stop solution was pipetted in each well and the plate red at 570 nm at VictorTM X4 (Perkin Elmer). IL-1β ELISA kit (BD Biosciences, San Diego, California, USA, 557953) was used following manufacturer’s instructions. Briefly, 96 well flat-bottom plates were coated with 100 μL/well of capture antibody and incubated overnight at 4°C. The day after, washes were performed and blocking solution was added. Samples and standards were pipetted (50 μL/well) to the appropriate wells, immediately followed by IL-1β detection antibody and incubated for 2 hours at RT. After washes, streptavidin-HRP was added in each well and incubated for 30 minutes at RT, then 100 µl/well of TMB were added for 15 minutes at RT. Finally, 50 μL/well of stop solution was pipetted in each well and the plate red at 450 nm at VictorTM X4. Col11a1 kit (FineTest, Wuhan, China, EH2859) was used following manufacturer’s instructions. Briefly, samples and standards were pipetted (100 μL/well) to the appropriate wells of a 96 well pre-coated flat-bottom plates for 90 min at 37°C. After washes, biotin-labelled detection antibody was incubated for 60 minutes at 37°C. Then, 100 μL/well SABC working solution was added and incubated for 30 minutes at 37°C. Finally, 100 µl/well of TMB were added for 20 minutes at 37°C, then 50 μL/well of stop solution was pipetted in each well and the plate red at 450 nm at VictorTM X4.

**Western Blot**

Treated fibroblasts were collected after 7 days and homogenized in RIPA buffer (supplemented with protease inhibitor and phosphatase inhibitor). Protein extraction was performed by applying 5 cycles of sonication and cooling on ice. Protein concentration was determined using the BCA assay (Life Technologies). Then, 20 μg protein/sample was loaded in SDS-PAGE and analysed by WB using specific primary antibodies against αSMA (Dako, M0851), MAGP-2/MFAP5 (Novus, NBP1-82803), GAPDH (BioRad, MCA4740), phospho-NF-κB p65 (Ser536, Cell signaling, 3033), and FAP (Cell Signalling, E1V9V) followed by appropriate secondary antibodies anti-rabbit-HRP or anti-mouse-HRP (BioRad, 170-6515 and 170-6516).

**Original, uncropped Western Blot images with unadjusted contrast and bands of protein markers (reported as kDa).**

Figure 1

SMA 42 kDa


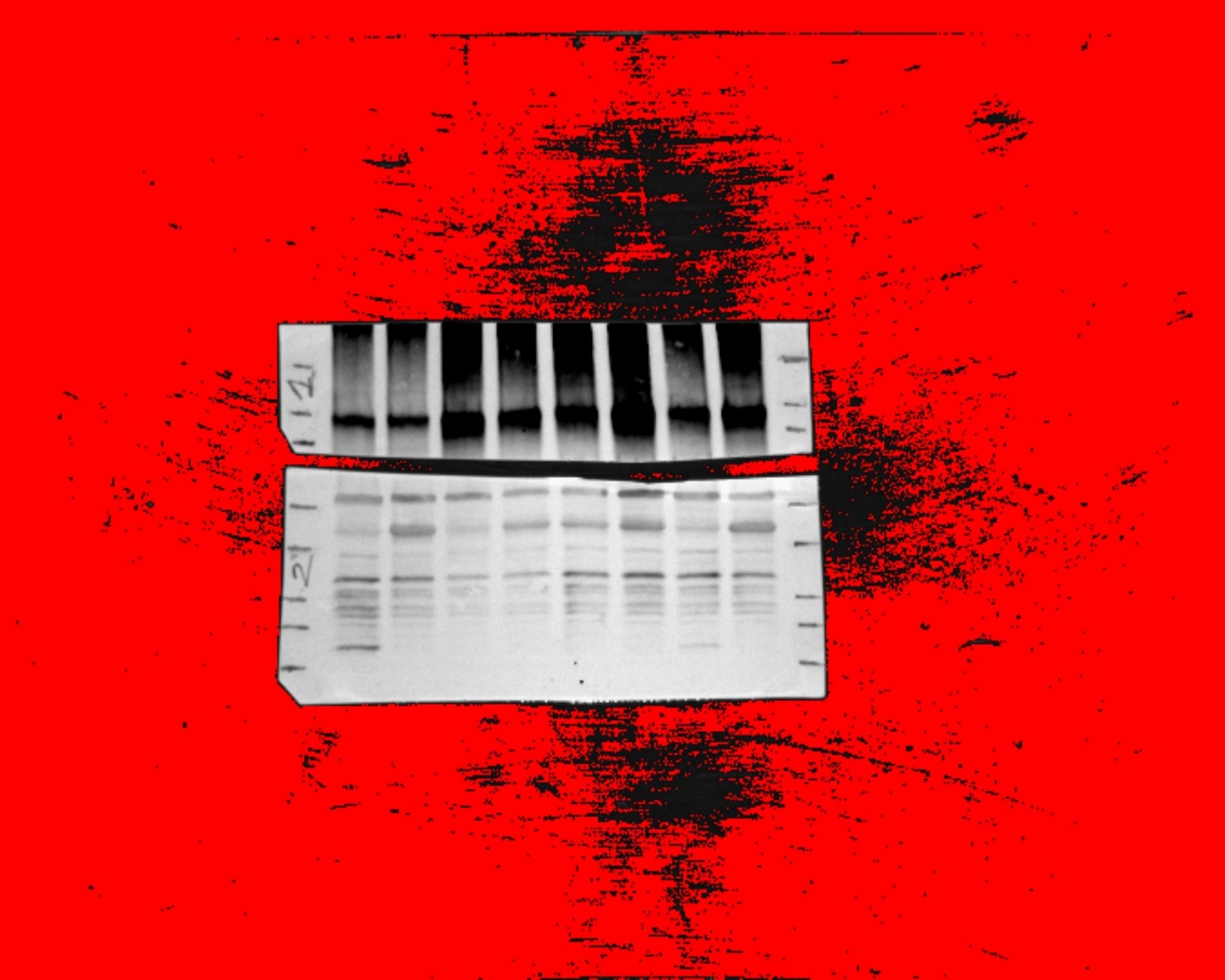


37

50

25

FAP 90 kDa

75

100


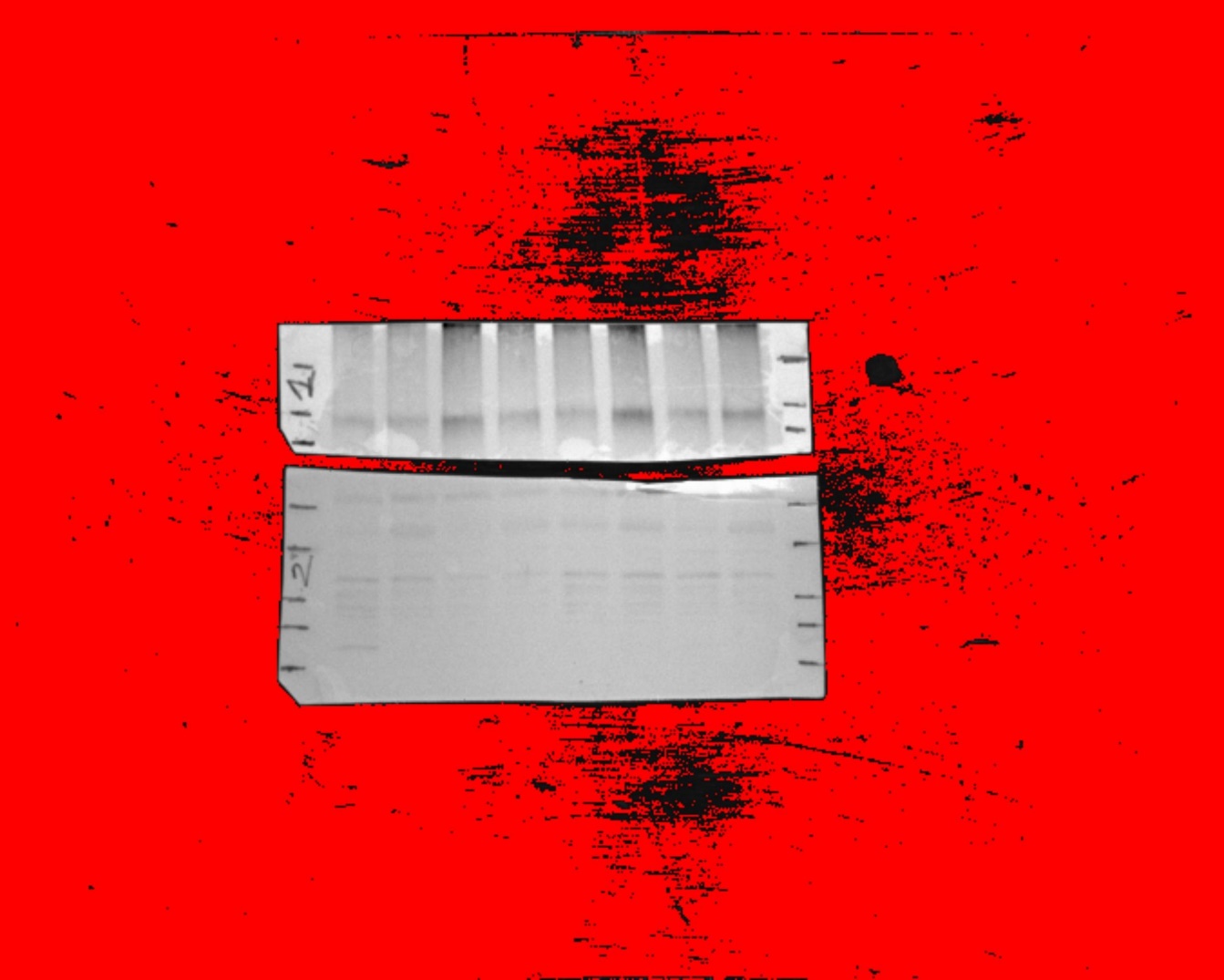


GAPDH 36 kDa


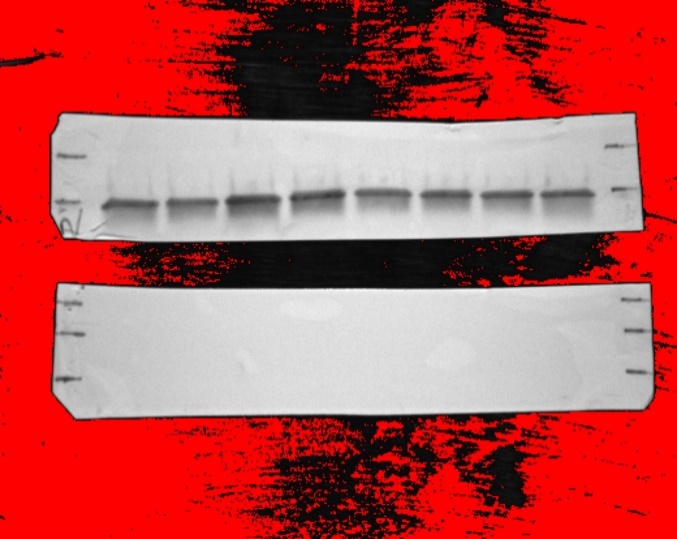


50

37

Figure S1

GAPDH 36 kDa


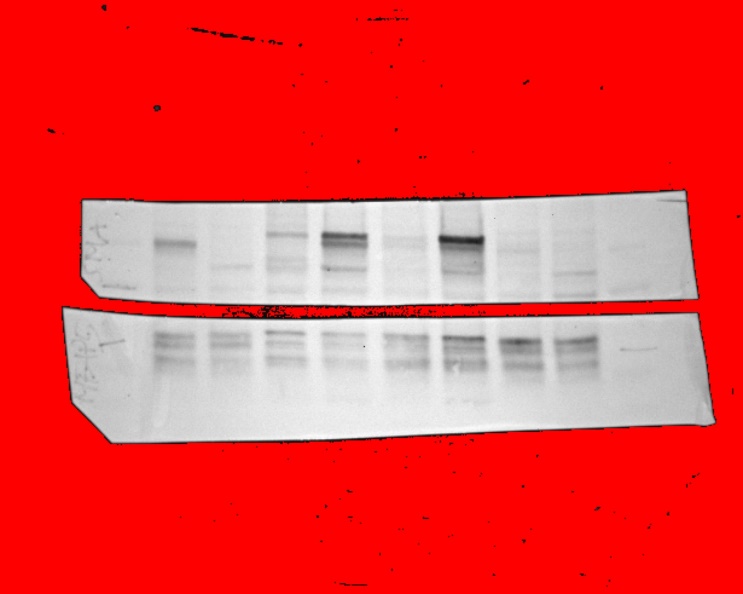


25

20


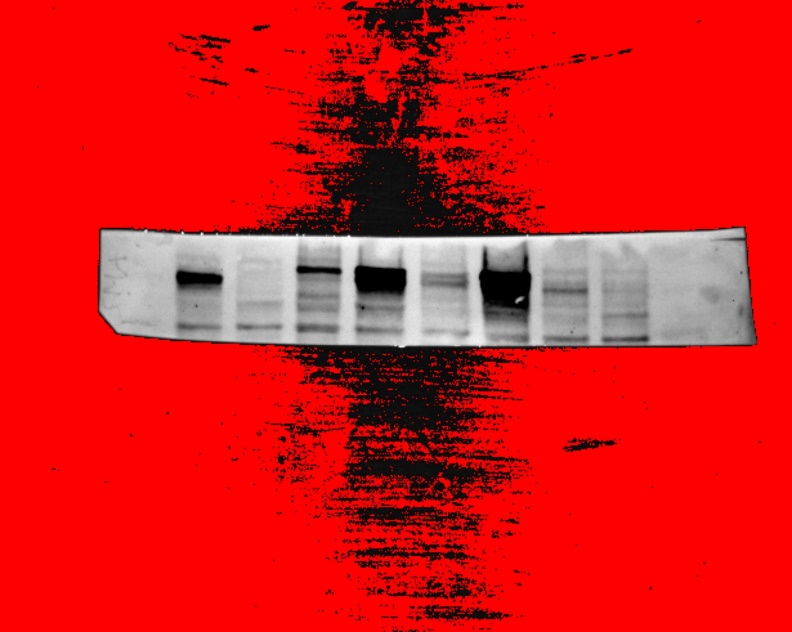


37

50

MFAP5 20-25 kDa

Figure S4

Phospho-NF-kB 65 kDa


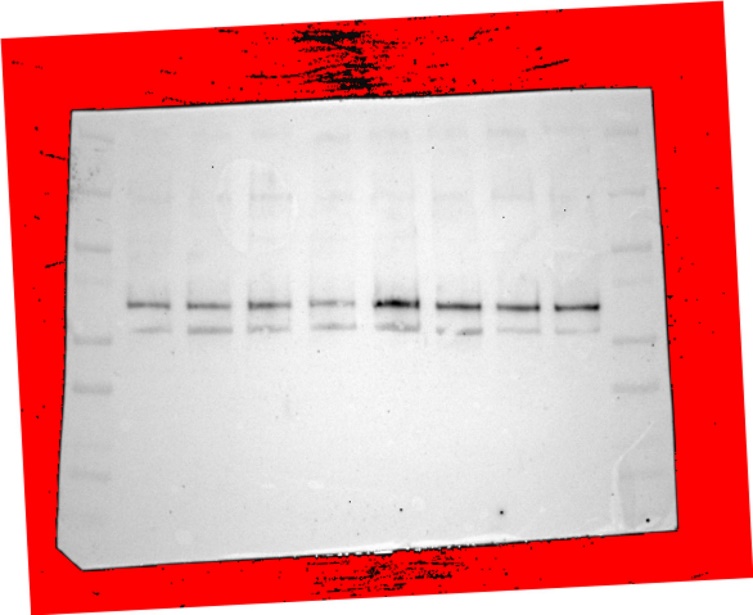


75

50

GAPDH 36 kDa


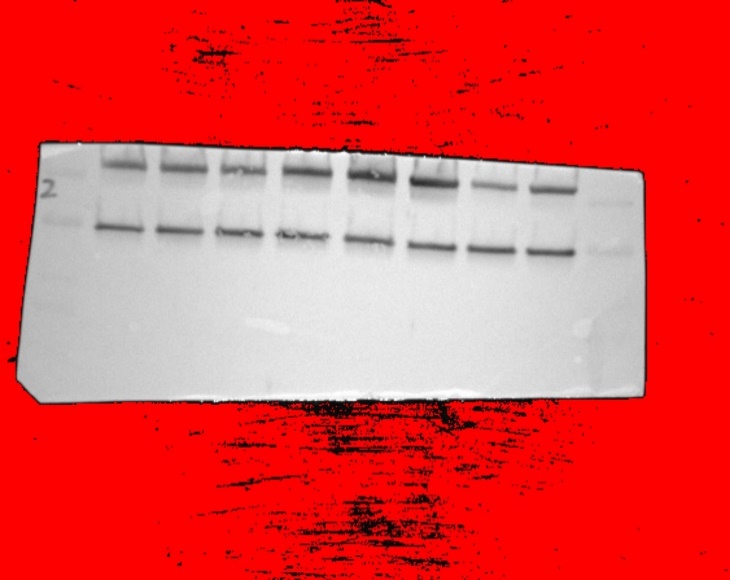


50

37
